# Activatable biomimetic probe with aggregation-induced emission characteristics for non-invasive monitoring of allograft rejection

**DOI:** 10.7150/thno.110866

**Published:** 2025-05-30

**Authors:** Mengdan Ding, Fang He, Shuangze Han, Wuqi Zhou, Tian Huang, Nan Cui, Yuanting Quan, Wenqu Li, Wenyuan Wang, Tang Gao, Mingxing Xie, Li Zhang

**Affiliations:** 1 Department of Ultrasound Medicine, Union Hospital, Tongji Medical College, Huazhong University of Science and Technology, Wuhan 430022, China;; 2 Hubei Province Clinical Research Center for Medical Imaging, Wuhan 430022, China;; 3 Hubei Province Key Laboratory of Molecular Imaging, Wuhan 430022, China.

**Keywords:** macrophages, transplant rejection, activatable probe, fluorescence imaging, aggregation-Induced emission.

## Abstract

**Abstract**: Background: Allograft rejection remains a major barrier to the long-term success of organ transplantation. The current gold standard for diagnosis-tissue biopsy is invasive and carries inherent risks, including sampling errors, procedural complications, and high costs. There is a pressing need for an efficient, non-invasive strategy for the early detection and monitoring of transplant rejection.

Methods: We developed a macrophage-targeted, activatable imaging probe (**MTBPB/GPs**) by encapsulating the H₂O₂-responsive aggregation-induced emission (AIE) molecule** MTBPB** into glucan particles (GPs) via electrostatic and hydrophobic interactions. The probe's responsiveness to H₂O₂ was characterized using UV-vis and fluorescence spectroscopy. Biocompatibility was evaluated through hemolysis assays, immunogenicity testing, biochemical analysis, and histopathology. Macrophage polarization and probe specificity were assessed using confocal laser scanning microscopy (CLSM), flow cytometry (FCM), and ELISA. A murine dorsal skin transplantation model was established to dynamically monitor graft rejection and the therapeutic efficacy of FK506, using *in vivo* fluorescence imaging at postoperative days (POD) 1, 3, 5, and 7. Pathological validation was performed via H&E staining and immunofluorescence.

Results: **MTBPB/GPs** exhibited excellent biosafety, with low cytotoxicity, minimal hemolytic activity, low immunogenicity, and negligible organ toxicity. Upon oral administration, the fluorescence signal of **MTBPB/GPs** was selectively activated by M1 macrophages, enabling early and sensitive detection of transplant rejection. Moreover, a single oral dose allowed real-time tracking of immunosuppressive therapy with FK506**.**

Conclusion: **MTBPB/GPs** represent a promising non-invasive platform for early diagnosis and longitudinal monitoring of transplant rejection and therapeutic response, with strong translational potential in solid organ transplantation.

## Introduction

Organ transplantation remains the most effective and widely adopted therapeutic strategy for patients with end-stage organ failure 1, 2. Despite substantial advancements in surgical techniques and immunosuppressive regimens, transplant rejection (TR) continues to be the leading cause of graft failure 3, 4. Currently, the clinical gold standard for diagnosing rejection is histopathological biopsy 5, 6. However, biopsy is inherently invasive, posing risks such as bleeding, infection, and sampling errors, and may lead to false-negative results 7, 8. Therefore, the development of noninvasive approaches for the early detection of TR is urgently needed and holds substantial clinical value for improving graft prognosis and patient outcomes.

Given the limitations of current diagnostic approaches, there is growing interest in identifying early cellular and molecular events that contribute to transplant rejection. Among these, macrophages have emerged as key immune effectors in graft outcomes. Recent advances in transplant immunology have highlighted the functional heterogeneity of macrophages, particularly the distinct roles of M1 and M2 phenotypes in graft rejection. M1 macrophages, also known as classically activated macrophages, are induced by pro-inflammatory stimuli such as interferon-γ (IFN-γ) and lipopolysaccharide (LPS) 9, 10. These cells play a pivotal role in the early stages of acute rejection by secreting high levels of pro-inflammatory cytokines (e.g., TNF-α, IL-6) and reactive oxygen species (e.g., H₂O₂), thereby exacerbating graft inflammation and contributing to tissue injury 11, 12. In contrast, M2 macrophages are alternatively activated and are primarily involved in anti-inflammatory responses, tissue remodeling, and graft accommodation during the resolution or chronic phases of transplantation 13.

Clinical studies have further demonstrated that macrophage infiltration in biopsy samples correlates with transplant prognosis 14, 15. Notably, in renal transplant recipients with graft dysfunction, over 70% of infiltrating macrophages have been identified as the M1 subtype 16. Moreover, M1 macrophage accumulation has been implicated in a variety of adverse post-transplant events, including antibody-mediated rejection, ischemia-reperfusion injury, and chronic fibrosis 17-19. Collectively, these findings suggest that M1 macrophage infiltration serves as a critical immunological marker and a potential early indicator of acute transplant rejection.

Several molecular imaging strategies have been developed to monitor macrophage activity for the early diagnosis of graft rejection 20-23. For instance, macrophages readily internalize ¹⁸F-fluorodeoxy-glucose (¹⁸F-FDG), enabling positron emission tomography (PET) to visualize immune cell infiltration at transplant sites 24. Similarly, superparamagnetic iron oxide nanoparticles (SPIONs) serve as effective magnetic resonance imaging (MRI) contrast agents that accumulate within macrophages, providing non-invasive indicators of rejection severity 20, 21, 25. However, these techniques generally lack active targeting specificity and are often limited by background interference due to passive uptake and nonspecific biodistribution.

Beyond conventional PET and MRI probes, advances in nanomedicine have introduced a wide range of non-invasive imaging platforms with improved resolution, depth, and molecular specificity. These include second near-infrared window (NIR-II) nanoprobes based on thulium or erbium complexes for deep-tissue and real-time imaging 26, 27; activatable fluorescent reporters that respond to enzymatic activity or inflammatory stimuli 28; and multifunctional theranostic nanoplatforms capable of simultaneous diagnosis and therapy 29, 30. Additionally, emerging photonic imaging technologies continue to enhance spatial resolution and signal-to-background ratios *in vivo* 31. While these approaches represent important progress, many still rely on intravenous administration and lack specificity for macrophage subtypes or inflammatory microenvironments.

Glucan particles (GPs), derived from yeast cell walls, were first reported by Ostroff et al. as oral delivery vehicles that mimic the natural infection pathway of yeast 32. Upon oral administration, GPs are transcytosed by M cells in the intestinal epithelium and delivered to gut-associated lymphoid tissues, where they are preferentially phagocytosed by macrophages 33. This foundational work paved the way for macrophage-targeted disease diagnosis and therapy via oral delivery. Building on this concept, Zhang et al. successfully encapsulated quantum dots and paclitaxel into GPs for the oral treatment and imaging of subcutaneous breast cancer xenografts 34. Further extending this strategy, Sung and colleagues developed doxorubicin (DOX)-loaded GPs that not only crossed the intestinal barrier but also enabled macrophage-mediated delivery across the blood-brain barrier, achieving oral “gut-to-brain” glioblastoma targeting 35. These studies position GPs as promising biomimetic carriers for oral macrophage targeting. However, direct conjugation of imaging agents or therapeutics to GPs remains technically challenging. Most approaches require pre-formulation of positively charged nanoparticles or polymeric encapsulation, adding complexity to probe design and limiting scalability.

Fluorescence imaging, owing to its high spatiotemporal resolution, noninvasiveness, and ease of use, has been widely applied for real-time tracking of macrophage dynamics *in vivo* 36. Near-infrared (NIR) fluorophores, especially in the 650-900 nm range, offer improved tissue penetration and reduced autofluorescence, making them well-suited for deep-tissue imaging 37. However, many traditional NIR probes suffer from aggregation-caused quenching (ACQ) when used at high concentrations or when incorporated into delivery platforms, significantly impairing fluorescence intensity and imaging sensitivity 38. Aggregation-induced emission (AIE) luminogens overcome this limitation by exhibiting enhanced fluorescence in the aggregated state. In our previous work, we demonstrated that cationic AIE molecules could stably bind to GPs in aqueous media 39. Based on this platform, we constructed AIE-based biomimetic probes that were orally administered, phagocytosed by macrophages, and trafficked to allograft sites for the detection of transplant rejection. However, the probes exhibited a fluorescence “always-on” behavior, generating background signals in non-rejecting tissues and thereby reducing diagnostic contrast.

To improve imaging specificity, activatable probes have emerged as a preferred solution. These probes remain in an “off” state under physiological conditions and are selectively turned “on” upon encountering disease-specific stimuli such as enzymes, pH shifts, or oxidative stress 40, 41. In the context of transplant rejection, macrophages infiltrating the graft undergo polarization toward the pro-inflammatory M1 phenotype under the influence of cytokines such as TNF-α and IL-2 42-44. M1 macrophages significantly upregulate inducible nitric oxide synthase (iNOS) and NADPH oxidase, leading to elevated production of reactive oxygen species (ROS), particularly hydrogen peroxide (H₂O₂), a key mediator of oxidative stress and tissue injury 45-49.

**Scheme 1:**
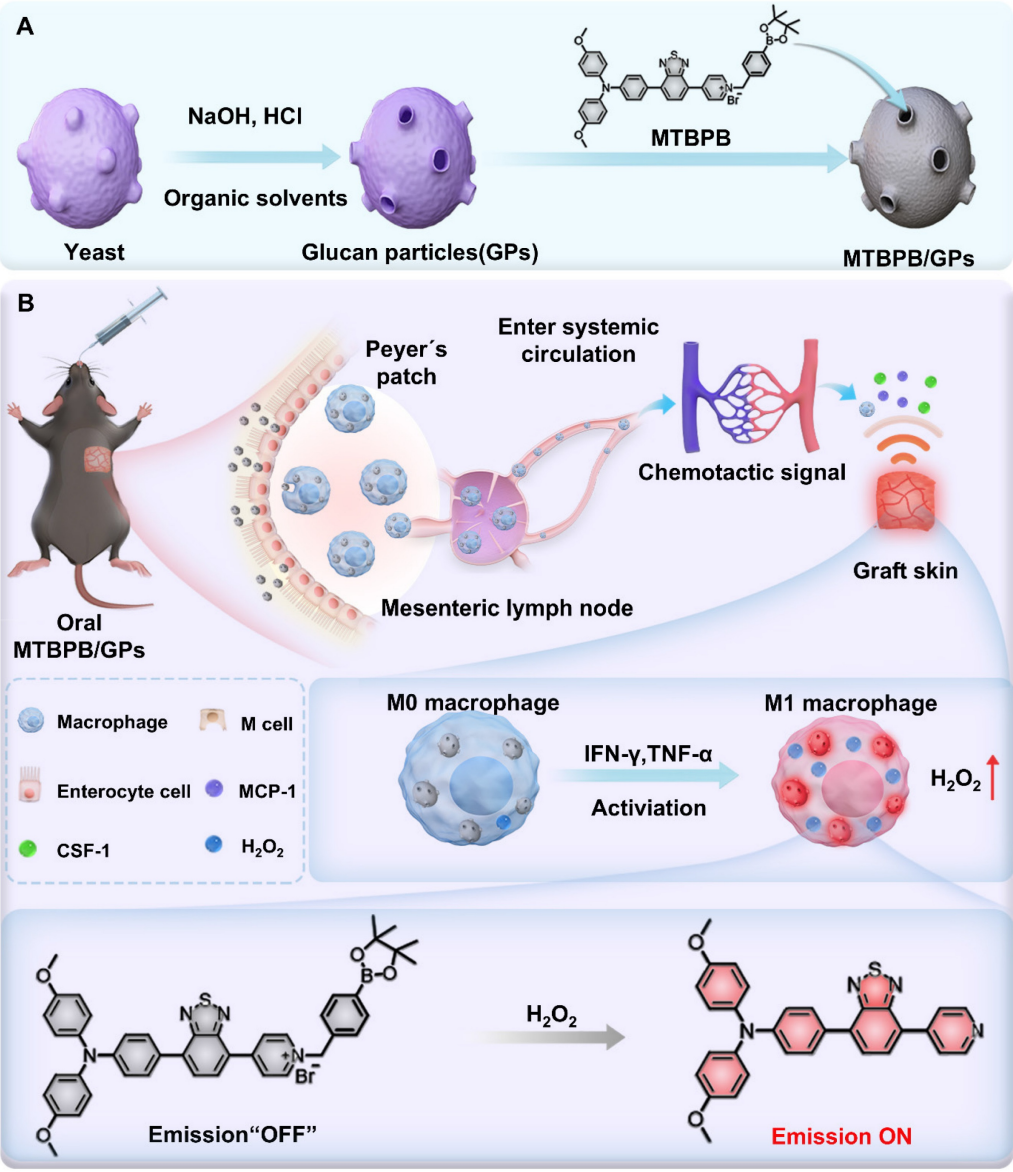
(A) Preparation of the biomimetic probe MTBPB/GPs. (B) In an allogeneic mouse transplant model, orally administered MTBPB/GPs specifically target macrophages. Guided by chemokines, macrophages loaded with MTBPB/GPs migrate to the graft site. When stimulated by inflammatory cytokines, these macrophages generate elevated levels of H₂O₂, which activates the fluorescence MTBPB/GPs, enabling the early detection of TR.

Given the central role of M1 macrophages in acute transplant rejection, we hypothesized that selectively imaging their activity through H₂O₂-responsive activation could provide a noninvasive and sensitive strategy for early diagnosis. To this end, we designed a biomimetic, activatable probe by encapsulating an H₂O₂-sensitive AIE molecule, **MTBPB**, into GPs (**Scheme 1A**). **MTBPB** contains a boronate ester moiety that reacts selectively with H₂O₂ 50, triggering a fluorescence turn-on response in oxidative inflammatory environments. Upon oral administration, **MTBPB/GPs** are taken up by intestinal macrophages and transported to the graft. In the presence of inflammatory stimuli, these macrophages polarize to the M1 phenotype, generating H₂O₂ that activates **MTBPB** fluorescence. This inflammation-dependent activation ensures minimal background fluorescence in non-rejecting tissues and enables precise visualization of rejection-specific macrophage infiltration (**Scheme 1B**). By integrating macrophage-mediated delivery with ROS-triggered activation, our system achieves high target specificity, low background interference, and fully noninvasive imaging. Thus, **MTBPB/GPs** offer a powerful platform for the early diagnosis and longitudinal monitoring of transplant rejection, with strong translational potential as a clinically viable alternative to invasive biopsy.

## Experimental methods

### Preparation of GPs

First, 13 g of Angel yeast was added to 200 mL of 1 M sodium hydroxide (NaOH) solution. The mixture was incubated in a water bath at 80 ^°^C with stirring for 1 h. After naturally cooling to room temperature, the solution was centrifuged at 2000 ×g for 15 min to collect the precipitate. The pH of the resulting solution was then adjusted to 4-5 using diluted hydrochloric acid (HCl), followed by incubation in a 60 ^°^C water bath for another 1 h. After cooling to room temperature, the precipitate was washed 3 times with double-distilled water. Subsequently, the precipitate was washed 4 times with 200 mL isopropanol and twice with 200 mL acetone. The final precipitate was collected by centrifugation (2000 ×g, 15 min) and freeze-dried for two days to obtain the GPs powder.

### Preparation of MTBPB/GPs

The **MTBPB** molecule was synthesized by modifying a borate ester reaction site on the AIE molecular probe according to the previous research method of our group 39, 51. Next, 10 mg of GPs were dissolved in 1 mL of PBS solution and incubated for 30 min at 37 ^°^C. Subsequently, 100 μL of 1 mM **MTBPB** ethanol solution was added to the reaction system and continued to be incubated at 37 ^°^C protected from light for 6 h. Subsequently, the precipitate was collected by centrifugation (3000 rpm, 2 min) and washed 3 times with double-distilled water to obtain **MTBPB/GPs** that was stored at 4 ^°^C protected from light.

### Characterization of MTBPB/GPs

Yeast microcapsules (YM), GPs and **MTBPB/GPs** were dissolved using double-distilled water, sonicated for 30 s, and then 200 μL was taken and added dropwise to the wafer. The wafers were dried in an oven at 60 ^°^C for 3 h and subsequently fixed on the sample stage by conductive adhesive. After spraying platinum for 5 min, the samples were removed and observed using a field emission scanning electron microscope (SEM). In addition, 10 μL of YM, GPs and **MTBPB/GPs** solutions were taken and added dropwise to a 400-mesh copper mesh, which was allowed to dry naturally, and then the morphological characteristics were observed by transmission electron microscopy (TEM).

The 5 mL of YM, GPs and **MTBPB/GPs** solutions were configured using double-distilled water. A Malvern particle size analyzer was used for detection, and each sample was repeated 3 times and averaged. Small quantities of **MTBPB**, GPs and **MTBPB/GPs** powders were taken separately and the major functional groups in the range of 4000 to 500 cm⁻¹ were measured using FT-IR. In addition, XRD spectra of GPs and **MTBPB/GPs** powders were measured with the scanning range set from 2θ = 3^°^ to 2θ = 50^°^.

### The stability of MTBPB/GPs

To assess the colloidal stability of **MTBPB/GPs**, the samples were stored at 4 ^°^C, and the encapsulation efficiency and particle size were measured on days 1, 3 and 7. To simulate the highly acidic environment of the stomach, **MTBPB/GPs** were exposed to a simulated gastric fluid (pH 1.2), and the encapsulation efficiency was measured at different time points (10 min, 30 min, 1 h, 2 h). **MTBPB/GPs** were solubilized in anhydrous acetonitrile. After ultrasonic for 10 min, the **MTBPB** concentration in the supernatant was quantified, and the Loading Efficiency (LE) and Loading Content (LC) values were calculated.

### The ROS responsiveness and sensitivity of MTBPB

The 10 μL of 1 mM **MTBPB** was dissolved in 1 mL of PBS. The experiment was divided into 2 groups: one group was added with 5 μM H₂O₂ and the other group was added with an equal amount of PBS. The absorbance changes of the solution before and after the addition of H₂O₂ was measured using a UV spectrophotometer. The wavelength range was set at 200-670 nm, and the scanning step was 1 nm. Next, the fluorescence absorption spectra of the solutions with different concentrations of H₂O₂ (0-6 μM) were measured using a fluorescence spectrophotometer. The excitation wavelength of the fluorescence spectrophotometer was set at 520 nm, the excitation slit at 3.0 nm, the emission slit at 5.0 nm, and the scanning speed was set at 600 nm/min.

In addition, 10 μL of 1 mM **MTBPB** was dissolved in different concentrations of water/DMSO (0-95%) mixtures, and the fluorescence absorption spectra were measured using the same instrumental parameters. For dynamic monitoring, 10 μL of 1 mM **MTBPB** solution was taken and 5 μM H₂O₂ or an equal amount of PBS was added to each solution. The parameters of the fluorescence spectrophotometer were set to an excitation wavelength of 520 nm, and the fluorescence intensity at the absorption wavelength of 670 nm was collected. The fluorescence intensity was recorded every 20 s for 6 h.

### The selectivity of MTBPB

The following chemicals were dissolved in PBS solution: glucose (Glu), cysteine (Cys), glutathione (GSH), nitric oxide (NO), acetic acid (CH₃COOH), zinc chloride (ZnCl₂), copper chloride (CuCl₂), magnesium sulfate (MgSO₄), sodium chloride (NaCl), potassium chloride (KCl), peroxynitrite (ONOO^-^), hydroxyl radical (▪OH) and hydrogen peroxide (H₂O₂). The 10 μL of 1 mM **MTBPB** was dissolved in the above mixture and the fluorescence intensity at the absorption wavelength of 670 nm was measured using the same instrumental parameters.

To study the effect of pH on the reaction, the pH of **MTBPB** solution was adjusted to 4-10 using HCl and NaOH. 5 μM H₂O₂ was added in equal amounts at each pH and the fluorescence intensity was measured using the same instrumental parameters.

Additionally, to investigate the emission spectra of **MTBPB** in different organic solvents, 10 μL of 1 mM **MTBPB** solution was dissolved in various organic solvents, and the UV absorption and fluorescence emission spectra were measured using the same instrumental parameters.

### Cell culture

The RAW264.7 macrophage cells were cultured in DMEM medium containing 10% fetal bovine serum (FBS), 100 U/mL penicillin, and 100 μg/mL streptomycin in a humidified atmosphere at 37 ^°^C with 5% CO₂. H9C2 and 293T cells were cultured under the same conditions as the macrophages. AML12 cells were cultured in MEM complete medium (MEM supplemented with 10% FBS, 2 mM L-glutamine, 100 U/mL penicillin, and 100 μg/mL streptomycin).

### Experimental animals

C57BL/6 and BALB/c mice (6-8 weeks old, 20-25 g) were purchased from Beijing Viton Lihua Co. Ltd. and housed in the Animal Breeding Centre of Tongji Medical College. All experimental procedures were in accordance with Laboratory Animal Ethics (No. 4197).

### Biosafety of MTBPB/GPs

Healthy C57BL/6 mice were randomly divided into healthy and **MTBPB/GPs** groups (n = 5). The **MTBPB/GPs** group received oral **MTBPB/GPs** (100 mg/kg) for 7 consecutive days. After 7 days, all mice were sacrificed, and peripheral blood was collected for hematology and evaluation of hepatic and renal function. Serum was analyzed for IgG and IgM levels to assess immunogenicity. The major organs were subsequently collected on days 1, 3, 7 and subjected to H&E staining.

### Cytotoxicity of MTBPB/GPs

A negative control group and an **MTBPB/GPs** group were established (n = 5). The negative control group was treated with DMEM medium only. Macrophages were incubated with **MTBPB/GPs** for 24 h at ratios of 1:20, 1:50, 1:100, and 1:200. Macrophage viability was assessed using the CCK-8 kit. The cytotoxicity of **MTBPB/GPs** on cardiomyocytes (H9C2), hepatocytes (AML12), and renal cells (293T) were also evaluated using the CCK-8 kit (n = 5).

### The phenotype of macrophage

The RAW264.7 cells (1 × 10^⁵^ per well) were seeded in 6-well plates and separated into two groups: LPS and control (n = 5). The LPS group was incubated with LPS (1 μg/mL) for 24 h, while the control group was treated with complete medium. Each group was incubated for 30 min with 10 μL of viability dye (FVS-AF700 channel) and washed 2 times with PBS. Then, 2 μL of F4/80 and CD86 antibodies were added to each group and incubated in the dark at 4 ^°^C for 40 min. Finally, the expression levels of F4/80 and CD86 were measured using flow cytometry (F4/80-BV421 and CD86-PE-Cy7 channels).

The RAW 264.7 cells (8 × 10^³^ per well) were seeded in 96-well plates, referring to the above grouping. The concentrations of TNF-α and IFN-γ in the supernatants, both before and after LPS stimulation, were quantified using an ELISA kit (n = 5).

The RAW264.7 cells (1 × 10^⁵^ per well) were seeded into 6-well plates and divided into two groups: IL-4 group and control group (n = 4). The IL-4 group was cultured with IL-4 (500 ng/mL) for 48 h, while the control group was treated with complete medium. Each group was incubated with 10 μL of viability dye (FVS-AF700 channel) for 30 min and then washed 2 times with PBS. Next, 2 μL of F4/80 antibody was added to each group, followed by incubation at 4 ^°^C in the dark for 40 min. After membrane rupture with permeabilization buffer for 1 h, 2 μL of CD206 antibody was added to each group and incubated at 4 ^°^C in the dark for another 40 min. Finally, the expression levels of F4/80 and CD206 were measured using flow cytometry (F4/80-BV421 and CD206-AF647 channels).

### Fluorescence activation of MTBPB/GPs in M1 macrophages

The RAW264.7 cells (1 × 10^⁵^ per well) were seeded into 6-well plates or confocal culture dishes and divided into 4 groups: M0, M1, M1 + NAC, and M2. The M0 group was left untreated, the M1 group was stimulated with 1 μg/mL LPS for 24 h, the M1 + NAC group was stimulated with 1 μg/mL LPS for 24 h, followed by the addition of 5 mM N-acetylcysteine (NAC) for an additional 24 h co-incubation, and the M2 group was stimulated with 500 ng/mL IL-4 for 48 h.

Cells were then incubated with diluted DCFH-DA (10 μM) at 37°C for 45 min, followed by 5 times washed with PBS (n = 4). ROS production was assessed using the ROS-FITC channel. After honest 3342 staining, fluorescence imaging was performed using a laser scanning confocal microscope (LSCM). The fluorescence signal was quantified by flow cytometry.

Following the same experimental groups as above, **MTBPB/GPs** were added to the cells, and they were cultured for an additional 24 h (n = 4). After honest 3342 staining, fluorescence intensity was observed using LSCM. Cells in the 6-well plates were washed 3 times with PBS to remove excess **MTBPB/GPs**, and fluorescence intensity was quantified by flow cytometry (E_x_ = 480 nm, E_m_ = 620 nm).

### Co-localization of MTBPB/GPs with M1 macrophage lysosomes

The RAW264.7 cells (1 × 10^⁵^ per well) were seeded into confocal culture dishes and incubated with LPS for 24 h. The cells were then co-incubated with **MTBPB/GPs** for an additional 24 h, followed by 3 times with PBS. Lysosomes were labeled by incubating the cells with a lysosomal green fluorescence probe (FITC) for 1 h, followed by 2 times washed with PBS. The cells were then incubated with 200 μL honest 3342 in the dark for 10 min, followed by 2 times washed with PBS. Co-localization of lysosomes and **MTBPB/GPs** was observed using LSCM.

### Endocytosis pathway of MTBPB/GPs by M1 macrophages

RAW264.7 cells (1 × 10^⁵^ cells per well) were seeded in 6-well plates, using LPS stimulus 24h. One group was pretreated with the laminarin (1 mg/mL) for 2 h, while the other group received no treatment (n = 4). **MTBPB/GPs** (1 × 10^⁵^ per well) were then added and incubated for 12 h at 37 ^°^C. Cell suspensions from both the control and **MTBPB/GPs** groups were collected, which phagocytosis rates were quantified by flow cytometry (E_x_ = 480 nm, E_m_ = 620 nm).

### Macrophage migratory activity following endocytosis of MTBPB/GPs

RAW264.7 cells (1 × 10^⁵^ per well) were inoculated into 6 wells and cultured for 12 h (n = 4), then **MTBPB/GPs** were added and cultured for another 12 h. The upper layer of the chamber was filled with 200 μL of serum-free medium. The lower layer was injected with 20% medium containing 10 ng of MCP-1. 12 h later, the cells in the upper layer were wiped off with a moistened cotton swab, and the cells on the back of the upper layer were stained with DAPI. Finally, 3 randomly selected fields of view were observed under the microscope and the number of cells was counted.

### MTBPB/GPs *in vivo* responsiveness

The 24 allograft mice were randomly assigned to 6 groups (n = 4). Small animal live imaging was conducted on the grafted skins at 0, 6, 12, 24, 36, and 48 h following oral **MTBPB/GPs** (200 μL, 0.8 mg/kg **MTBPB**) on postoperative day (POD) 6. Imaging was performed using a Perkin Elmer *IVIS Lumina* system (E_x_ = 480 nm, E_m_ = 620 nm). Regions of interest (ROI) were outlined for quantitative fluorescence intensity analysis.

### Fluorescence imaging of TR

Allograft and isograft mice were orally administered **MTBPB/GPs**. (n = 5) Whole-body imaging was performed using a small-animal *in vivo* imaging system at 36 h after oral administration on POD 0, 2, 4, and 6, respectively. Imaging was conducted with a Perkin Elmer *IVIS Lumina* system (E_x_ = 480 nm, E_m_ = 620 nm).

Under the same modeling conditions as described above, **MTBPB/GPs** were administered orally to both groups on POD 6 (n = 5). Mice were sacrificed 24 and 36 h after oral administration, and major organs were harvested from the groups, and then were performed using a small animal live imaging system.

### Immunosuppressive therapy efficacy evaluation in real time

An allogeneic mouse model was established with continuous daily subcutaneous injections of FK506 (3.0 mg/kg) or PBS, starting on the day of surgery and continuing until POD 7 (n = 5). Both group received oral **MTBPB/GPs** (n = 4) on the day of surgery, and *in vivo* imaging was carried out on POD 1, 3, 5 and 7 to monitor the real-time effectiveness of the immunosuppressive treatment (Perkin Elmer *IVIS Lumina*: E_x_ = 480 nm, E_m_ = 620 nm).

### *In vivo* transport mechanism of MTBPB/GPs

Allograft mice on POD6 were randomly assigned to two groups, one group was orally administered **MTBPB/GPs** and the other group was orally administered an equal amount of **MTBPB** (n = 5). *In vivo* imaging was performed on the 36th h after oral administration and quantitative fluorescence analysis was performed. After the mice were executed, the gastrointestinal tract was used to for ex vivo imaging. (Perkin Elmer *IVIS Lumina*: E_x_ = 480nm, E_m_ = 620nm).

Subsequently, allograft mice on POD 6were randomly divided into 5 groups (n = 4). The gastrointestinal tract, Peyer's lymph nodes, mesenteric lymph nodes, draining lymph nodes, and isolated skin of the mice were removed after necropsy. Ex vivo imaging was performed after oral administration of **MTBPB/GPs** and at 0, 6, 12, 24, and 36 h, respectively. Imaging parameters were consistent with those described above. At the same time points, the mesenteric lymph nodes, Peyer's patches, draining lymph nodes, and excised skin were harvested from the mice. The tissues were subjected to fluorescence sectioning, DAPI staining, and the examination of the spontaneous fluorescence of **MTBPB/GPs** to track the migration pathway. Chemokine levels in the grafted skin were semi-quantified in healthy mice, allografts and syngeneic grafts at POD3 and POD7 (n = 4). The mRNA expression levels of MCP-1 and CSF-1 were quantified using real-time PCR.

### Histopathology and immunofluorescence analysis of graft skin

Mice were randomly assigned to 2 groups: one group received BALB/c skin grafts on the back, and the other received C57 skin grafts (n = 4). Skin were collected from both groups on POD 1, 3, 5, and 7. Hematoxylin-eosin (H&E) staining was performed, and the graft histology was evaluated by professional pathologists. The histological assessment was quantified.

Specific staining of skin tissue nuclei, CD68, iNOS using DAPI, FITC and Cy3 dyes, respectively. 3 fields of view were randomly selected for semi-quantitative the count of pro-inflammatory macrophages by fluorescence microscopy. The histopathology and immunofluorescence were evaluated in mice after 7 consecutive days of FK506 or PBS treatment using the same methods (n = 4).

Allograft and isograft skin grafts were collected from the dorsal of mice. The tissues were digested for 1 h with collagenase and dispase (2 mg/mL), followed by termination of digestion and filtration through a 70 μm filter. Each group was incubated with 10 μL of viability dye (FVS-AF700 channel) for 30 min, then washed twice with PBS. Subsequently, 2 μL of F4/80 and CD86 antibodies were added to each group and incubated at 4 ^°^C in the dark for 40 min. Finally, the expression levels of F4/80 and CD86 were measured using flow cytometry (n = 4).

### Statistical Analysis

The results are presented as mean ± SD. Continuous data following a normal distribution were analyzed using one-way ANOVA. Unpaired t-tests were applied for comparisons between two groups. All analyses were conducted using GraphPad Prism version 9.0 and Origin 2020. Statistical significance was defined as ^*^P < 0.05,^ **^P < 0.01, ^***^P < 0.001, and ^****^P < 0.0001.

## Results and discussion

### Design and photophysical property of H_2_O_2_-responsive probe MTBPB

**Figure 1:**
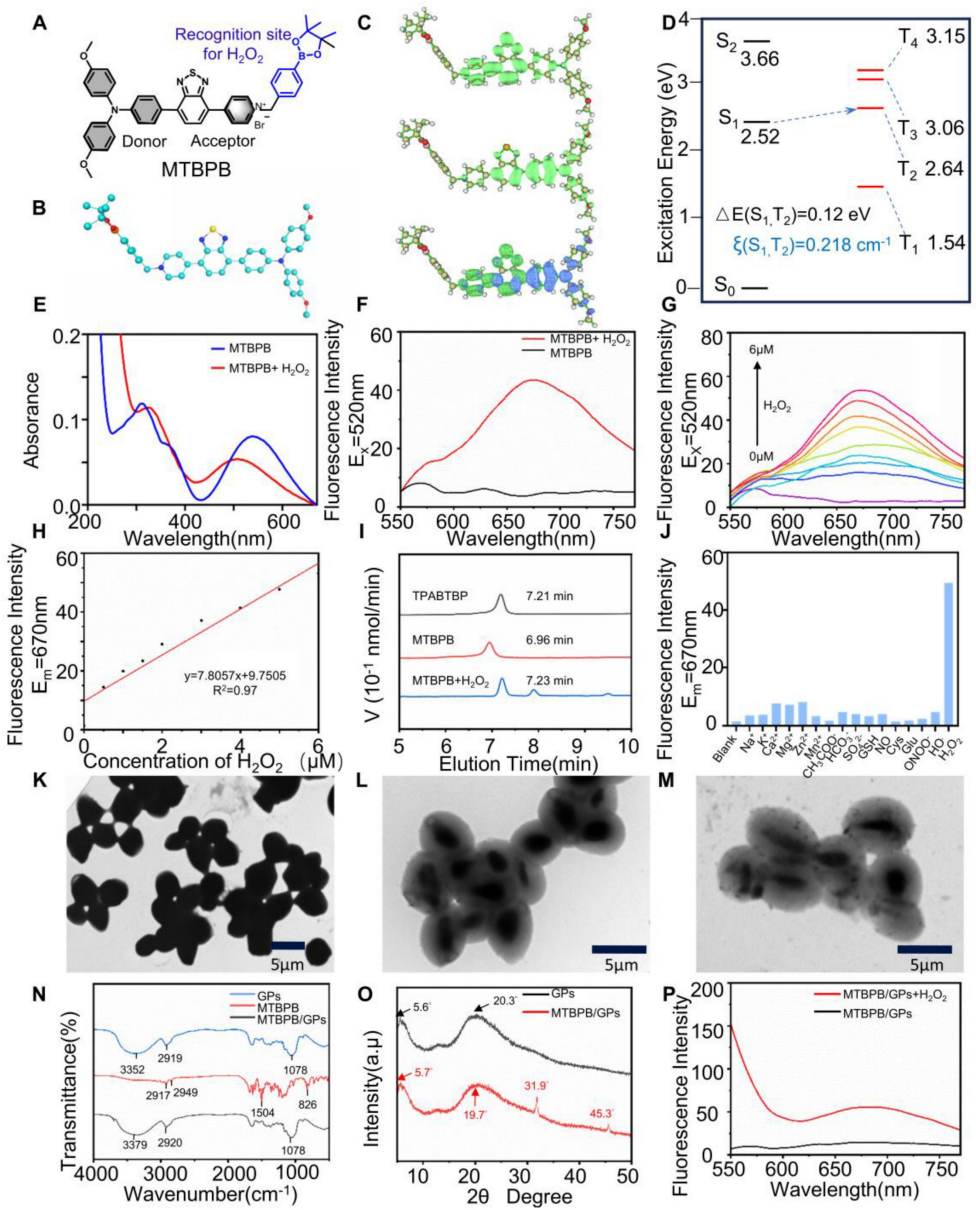
Characterization of probe **MTBPB/GPs** in response to H_2_O_2_. **(A)** structure and design of **MTBPB**. **(B)** The optimal configuration of the probe **MTBPB** in the ground state. **(C)** Electron-hole analysis of **MTBPB**. **(D)** The energy gap and associated spin-orbit coupling constants (ξ) of **MTBPB** were calculated based on the S0 structure using the CAM-B3LYP/def2-TZVP method. **(E)** The UV absorption and **(F)** fluorescence emission spectra of **MTBPB** (10 μM) with or without H_2_O_2_ (5 μM) in PBS. **(G)** Fluorescence emission spectra of **MTBPB** (10 μM) after incubation with different amounts of H_2_O_2_ (0-6 μM). **(H)** Correlation between the fluorescence intensity of **MTBPB** and the concentration of H_2_O_2_. **(I)** HPLC assays of **MTBPB** in the presence or absence of H_2_O_2_ and pure **TPABTBP**. **(J)** Fluorescence intensity (E_m_ = 670 nm) after mixture of **MTBPB** (10 μM) with the Na^+^, K^+^, Ca^2+^, Mg^2+^, Zn^2+^, CH_3_COO^-^, HCO_3_^-^, SO_4_^2-^, GSH, Cys, Glu, NO, ONOO^-^, ⋅OH and H_2_O_2_. **(K)** TEM image of yeast. **(L)** TEM image of GPs and **(M) MTBPB/GPs**. **(N)** FT-IR spectra of **MTBPB**, GPs, **MTBPB/GPs**. **(O)** XRD spectra of GPs, **MTBPB/GPs**. **(P)** Fluorescence emission spectra of **MTBPB/GPs** with or without H_2_O_2_ (5 μM) in PBS.

In our previous research, we successfully developed an advanced AIE molecule, **TPABTBP** 52. As in **Scheme S1**, the molecular structure of **TPABTBP** features typical donor-acceptor (D-A) configuration, with triphenylamine serving as the electron donor (D) and benzothiadiazol`e and pyridine as electron acceptors (A) (**Figure 1A,B**). The rotational flexibility between the donor and acceptor units endows the molecule with both aggregation-induced emission (AIE) and intramolecular charge transfer (ICT) properties. The compound **TPABTBP** underwent a salinization reaction with benzyl bromide borate, resulting in the formation of **MTBPB.** Designed with an amphiphilic structure and a positively charged pyridinium group, **MTBPB** effectively aggregates in the confined spaces of glucan particles through electrostatic interactions 39. The quinolinium unit's *p*-pinacolborylbenzyl group functions as a recognition moiety for H₂O₂ in biological systems. The molecular structure of **MTBPB** was analyzed using ^1^HNMR, ^13^CNMR, and spectrometry (**Figure S2-S9).**

First, the photophysical properties of **MTBPB** were studied. the AIE feature of **MTBPB** was studied in DMSO/water solutions with varying water concentrations (*f*_W_). As shown in **Figure S10A** and** 10B**, **MTBPB** exhibited weak fluorescence emission consistently as the water content increased from 0% to 95%. The weak fluorescence of **MTBPB** can be ascribed to the following factors: (1) The pyridinium group enhances the water solubility of** MTBPB**; (2) The incorporation of heavy halide ions (Br^-^) efficiently strengthens spin-orbit coupling (SOC), thereby promoting the efficient intersystem crossing (ISC) process and electron transfer 53; (3) The strong ICT effect makes it prone to intersystem crossing. To confirm the ICT characteristics of **MTBPB**, Multiwfn software was employed to perform hole-electron analysis, providing a comprehensive investigation of the electronic excitation features 54. As illustrated in the **Figure 1C**, the results revealed that the holes were predominantly localized in the electron-donating triphenylamine region, while the electrons are primarily distributed in the electron-accepting pyridinium group. The significant spatial separation between the hole and electron distributions strongly supports the conger conclusion that the **MTBPB** molecule exhibits pronounced ICT behavior. Strong ICT effects are typically accompanied by significant changes in the dipole moment, which may alter the nature of the excited state, bringing it closer to a triplet-state character (e.g., through the mixing of locally excited states). This, in turn, facilitates intersystem crossing (ISC) and contributes to fluorescence quenching. Therefore, we employed Time-Dependent Density Functional Theory (TD-DFT) calculations at the CAM-B3LYP/def2-TZVP level to calculate the energy gap between the singlet and triplet states of the **MTBPB** molecule. The energy transitions from S_1_ to T_2_ has been assumed due to the narrow energy gap of 0.12 eV. Moreover, the spin-orbit coupling (SOC) values for this transition was calculated to be 0.218 cm^-1^. The dense energy-level distributions and favorable SOC values provided a solid foundation for intersystem crossing (**Figure 1D**).

To further elucidate the photophysical behavior of **MTBPB** under different solvent environments, we investigated its absorption and emission charac-teristics in a series of organic solvents with varying polarity and proticity. As shown in **Figure S11A** and** S11B**, **MTBPB** exhibited a strong fluorescence emission peak at ~670 nm in non-polar solvents such as toluene, whereas its fluorescence intensity was significantly quenched in polar solvents such as DMF, CH₃OH, and H₂O. This behavior aligns with the classical AIE phenomenon, in which **MTBPB** molecules aggregate in low-polarity solvents, restricting intramolecular rotations and promoting radiative decay. In contrast, polar and protic solvents enhance solubility and allow for free intramolecular motion, thus favoring non-radiative pathways and quenching fluorescence emission. Additionally, the strong solvent-dependent photophysical response can be ascribed to the molecule's donor-acceptor (D-A) structure, comprising a triphenylamine donor and a pyridinium acceptor. In polar environments, this structure exhibits a pronounced intramolecular charge transfer (ICT) state, leading to charge-separated excited states and facilitating intersystem crossing (ISC) to triplet states. The presence of a bromide moiety further enhances spin-orbit coupling (SOC), reinforcing ISC and leading to further suppression of fluorescence in aqueous environments. These features confirm that **MTBPB** remains nearly non-emissive in physiological conditions but becomes selectively activated in environments with restricted solvation and elevated oxidative stress, such as those found in transplant rejection sites. Such AIE-ICT-ISC synergy ensures minimal background signal and high target-to-background contrast, making **MTBPB** an ideal candidate for activatable imaging of inflammatory microenvironments.

### Characterization of probe MTBPB in response to H₂O₂

We explored the fluorescent properties of **MTBPB** in the presence of H₂O₂. **MTBPB** displayed distinct absorptions at 310 and 540 nm, along with weak emission upon excitation at 520 nm. Upon the addition of H₂O₂ (5 μM), the absorption peak at 540 nm decreased in intensity, while a new peak emerged at 506 nm (**Figure 1E**). The observed blue shift in the absorption spectrum was due to the formation of **TPABTBP**, which decreased the intramolecular electron transfer. Furthermore, the fluorescence intensity at 670 nm increased markedly following the introduction of H₂O₂ (5 μM) (**Figure 1F**). Fluorescence titration studies showed a continuous amplification of the signal at 670 nm as the concentration of H₂O₂ was elevated (**Figure 1G**). Additionally, the fluorescence intensity at 670 nm demonstrated a robust linear relationship (R^2^ = 0.97) with the concentration of H₂O₂ (0-6.0 μM) (**Figure 1H**). According to the reported method, the detection limit of H₂O₂ for **MTBPB** was determined to be 0.11 μM 55, demonstrating the high sensitivity of **MTBPB** toward H₂O₂. To verify the formation of **TPABTBP** upon incubation of **MTBPB** with H₂O₂, we conducted high-performance liquid chromatography (HPLC) analysis. As shown in **Figure 1I**, the retention times for **TPABTBP** and** MTBPB** were 7.21 and 6.96 min, respectively. When exposed to H₂O₂ (5 μM), a new peak at 7.23 min with a mass-to-charge ratio (m/z) of 517.1689, confirming that the probe underwent oxidation-induced cleavage of the *p*-boratebenzyl group to generate **TPABTBP** (**Figure S12**).

Next, to determine the optimal reaction time for H₂O₂ detection, time-dependent absorption and fluorescence emission experiments were conducted using **MTBPB** in the presence of 5 μM H₂O₂ in PBS buffer (10.0 mM, pH 7.4, containing 5% DMSO). As shown in **Figure S10C**, in the absence of H₂O₂, the fluorescence intensity remains relatively stable over time, showing minimal variation. However, upon the introduction of 5 μM H₂O₂, a noticeable increase in fluorescence intensity is observed reached equilibrium within 6 h. The appropriate reaction rate between **MTBPB** and H₂O₂ ensures that the probe remains stable and unreactive in the background conditions of the gastrointestinal tract, reducing interference from low levels of H₂O₂. At the same time, it maintains high sensitivity and specificity toward higher H₂O₂ concentrations found in allograft rejection. The effect of pH on the **MTBPB** probe's ability to detect H₂O₂ was also explored. As shown in **Figure S10D**, the probe exhibits negligible fluorescence between pH 4 and 10. However, upon the addition of 5 μM H₂O₂, the **MTBPB** fluorescence signal was activated and enhanced within the pH range of 7-10. Since the reaction between **MTBPB** and H₂O₂ was a nucleophilic substitution process, these results indicated that highly acidic conditions were not conducive to the nucleophilic substitution, while the probe exhibited strong fluorescence respon-siveness under physiological pH conditions. To evaluate the selectivity of the **MTBPB** probe and exclude interference from other molecules and ions present in biological systems, a range of including metal cations (Na^+^, K^+^, Ca^2+^, Mg^2+^, Zn^2+^), ainions (CH_3_COO^-^, HCO_3_^-^, SO_4_^2-^ ), as well as biothiols such as GSH, Cys, Glu and other ROS like NO, peroxynitrite (ONOO^-^), hydroxyl radical (⋅OH). As shown in** Figure 1J,** the results demonstrated that the probe** MTBPB** produced negligible fluorescence when reacted with these potential interferents. In contrast, a pronounced fluorescence response was detected solely in the presence of H₂O₂. This selective fluorescence activation confirmed that the **MTBPB** probe displayed high specificity for H₂O₂ detection, with minimal cross-reactivity to other commonly encountered molecules or ROS.

### Characterization activatable biomimetic probe MTBPB/GPs

We synthesized glucan particles (GPs) using an acid-base organic solvent extraction method, followed by the encapsulation of the cationic H₂O₂-responsive AIE molecule MTBPB via electrostatic and hydrophobic interactions 32. Owing to the hollow and porous architecture of GPs, **MTBPB** is hypothesized to be effectively retained within the internal β-glucan matrix. Dynamic light scattering (DLS) analysis revealed that the average hydrodynamic diameter of **MTBPB/GPs** was approximately 3847 nm, which is comparable to native GPs but smaller than intact yeast shells (**Figure S13A**). The zeta potential of **MTBPB/GPs** was slightly negative (-4.8 mV), closely matching that of GPs and significantly reduced compared to the highly cationic **MTBPB** precursor (**Figure S13B**). This neutral surface charge is advantageous for reducing nonspecific protein adsorption and enhancing *in vivo* biocompatibility. Compared to the smooth surface of intact yeast (**Figure S14A**), the GPs exhibited a roughened exterior with pronounced wrinkles and pores measuring approximately 500-600 nm in diameter, consistent with the presence of yeast bud scars 35, 51, 56 (**Figure S14B**). Transmission electron microscopy further confirmed the successful formation of spherical, hollow particles and the efficient internalization of **MTBPB** within GPs (**Figure 1K-M**). The encapsulation efficiency of **MTBPB** reached 99.2%, with a drug loading content of 0.7%, supporting the strong affinity between **MTBPB** and the inner cavity of the GPs and validating the robustness of the loading strategy.

To further explore the interaction between **MTBPB** and GPs, FT-IR spectroscopy and XRD analyses were performed. In the FT-IR spectrum of GPs and **MTBPB/GPs (Figure 1N**), the peak at 1078 cm⁻¹ was attributed to the polysaccharide component. The peak at 1078 cm was ascribed to the polysaccharide component, while the one at 2919 cm⁻¹ was linked to the vibration of the -CH- groups. Additionally, the peak at 3352 cm⁻¹ corresponded to the stretching vibration of -OH groups. **MTBPB** exhibited alkyl C-H stretching vibrations at 2917 and 2949 cm⁻¹, along with the C=C stretching vibration of the aromatic ring at 1504 cm⁻¹. A peak between 700-900 cm⁻¹ range was attributed to the C-H bending vibrations on the benzene ring. In the **MTBPB/GPs** spectrum, the absence of the C-H bending peaks of the benzene ring and the C=C stretching vibration of the aromatic ring indicated the successful encapsulation of **MTBPB** within the GPs cavity. In the XRD spectra of GPs (**Figure 1O**), a diffraction peak at 5.6^°^ verified the existence of the triple helical configuration of β-glucan 57. Notably, the XRD spectrum of** MTBPB/GPs** showed distinct crystalline peaks at 31.9^°^, and 45.3^°^, suggesting that** MTBPB** formed crystalline states within the GPs. To further confirm the H₂O₂-activated properties of **MTBPB/GPs**, we anlyzed the fluorescence emission spectra of **MTBPB/GPs** before and after exposure to H₂O₂. As depicted in** Figure 1P** , **MTBPB/GPs** exhibited weak fluorescence in the absence of H₂O₂. Upon addition of H₂O₂, a significant fluorescence enhancement was observed at 670 nm, indicating that H₂O₂ effectively activated the **MTBPB/GPs**.

To evaluate the stability of **MTBPB/GPs** under both physiological and storage conditions, we investigated their behavior in simulated gastric fluid and under refrigerated storage. First, to assess acid stability, **MTBPB/GPs** were incubated in a buffer at pH 1.2 to mimic the gastric environment. After 2 h, the cumulative release of **MTBPB** remained below 25%, indicating minimal premature leakage and strong structural integrity under harsh gastrointestinal conditions (**Figure S15**). Next, the colloidal stability of **MTBPB/GPs** was assessed by monitoring particle size changes in PBS at 4 ^°^C over a 7-day period. The average hydrodynamic diameter remained consistently around 3.7 μm, with no evidence of aggregation or degradation (**Figure S16A**). In addition, loading efficiency (LE) remained above 95% on days 1, 3, and 7, suggesting strong encapsulation retention and negligible payload loss during refrigerated storage (**Figure S16B**). Together, these findings confirm the excellent structural and colloidal stability of **MTBPB/GPs** under both gastrointestinal and storage conditions, supporting their suitability for oral delivery and *in vivo* biomedical applications.

### Specific Targeting and Imaging of M1 Macrophages with MTBPB/GPs

**Figure 2:**
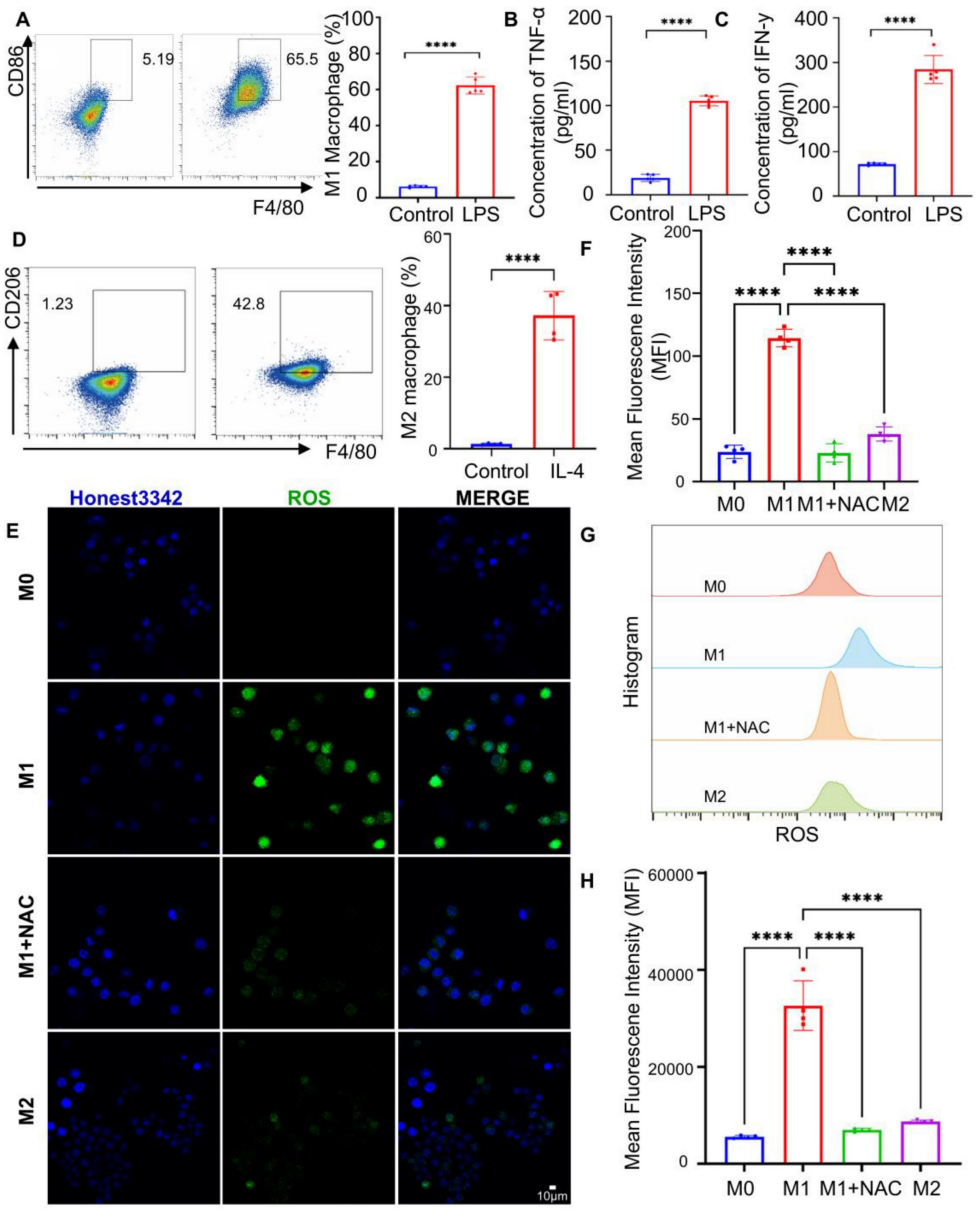
Macrophage polarization phenotype validation. **(A)** Representative images and quantitative analysis of CD86 expression in macrophages before and after LPS (1 µg/mL) stimulation by flow analysis (n = 5). **(B)** Measurement of TNF-α concentration and **(C)** IFN-γ in cell supernatant before and after LPS stimulation (n = 5). **(D)** Representative and quantitative analysis plots of CD206 measured by flow assay before and after 48 h stimulation with the addition of IL-4 (500 ng/ml) (n = 4). **(E)** Confocal representative images of intracellular ROS in M0, M1, M2, and M1 + NAC macrophages. **(F)** Fluorescence quantification plots (n = 4). **(G)** Flow representative images of intracellular ROS in M0, M1, M2, and M1 + NAC macrophages flow representative images. **(H)** flow quantitative analysis map (n = 4). ^***^P < 0.001, ^****^ P < 0.0001

Macrophages can be polarized into the pro-inflammatory M1 phenotype upon stimulation with LPS 58. This polarization is characterized by the upregulation of membrane CD86 and the secretion of pro-inflammatory cytokines such as TNF-α and IFN-γ 59, 60. To verify successful M1 induction, macrophages were treated with 1 μg/mL LPS for 24 h, and CD86 expression was analyzed via flow cytometry. Compared to unstimulated controls, CD86 levels increased nearly 10-fold (**Figure 2A**). ELISA analysis of the culture supernatant further confirmed polarization, revealing 5.5- and 3.9-fold increases in TNF-α and IFN-γ secretion, respectively (**Figure 2B, C**).

M1 macrophage polarization is also accompanied by elevated intracellular reactive oxygen species (ROS) levels 60, 61, which serve as key markers of oxidative stress during inflammation. To characterize ROS profiles across macrophage phenotypes, IL-4 stimulation was used to induce M2 polarization, as confirmed by CD206 upregulation via flow cytometry (**Figure 2D**). Confocal microscopy and flow cytometric analysis using ROS-sensitive fluorescent dyes revealed that M1 macrophages exhibited significantly higher ROS levels-4.8- and 5.8-fold increases relative to M0 controls, and 3.0- and 3.7-fold increases compared to M2 macrophages (**Figure 2E, F**). Treatment with the ROS scavenger N-acetylcysteine (NAC) substantially reduced ROS fluorescence intensity, confirming the oxidative profile of M1 macrophages (**Figure 2G, H**). These results collectively validate the successful induction of M1 polarization and underscore the distinct ROS-rich microenvironment associated with M1 macrophages.

**Figure 3:**
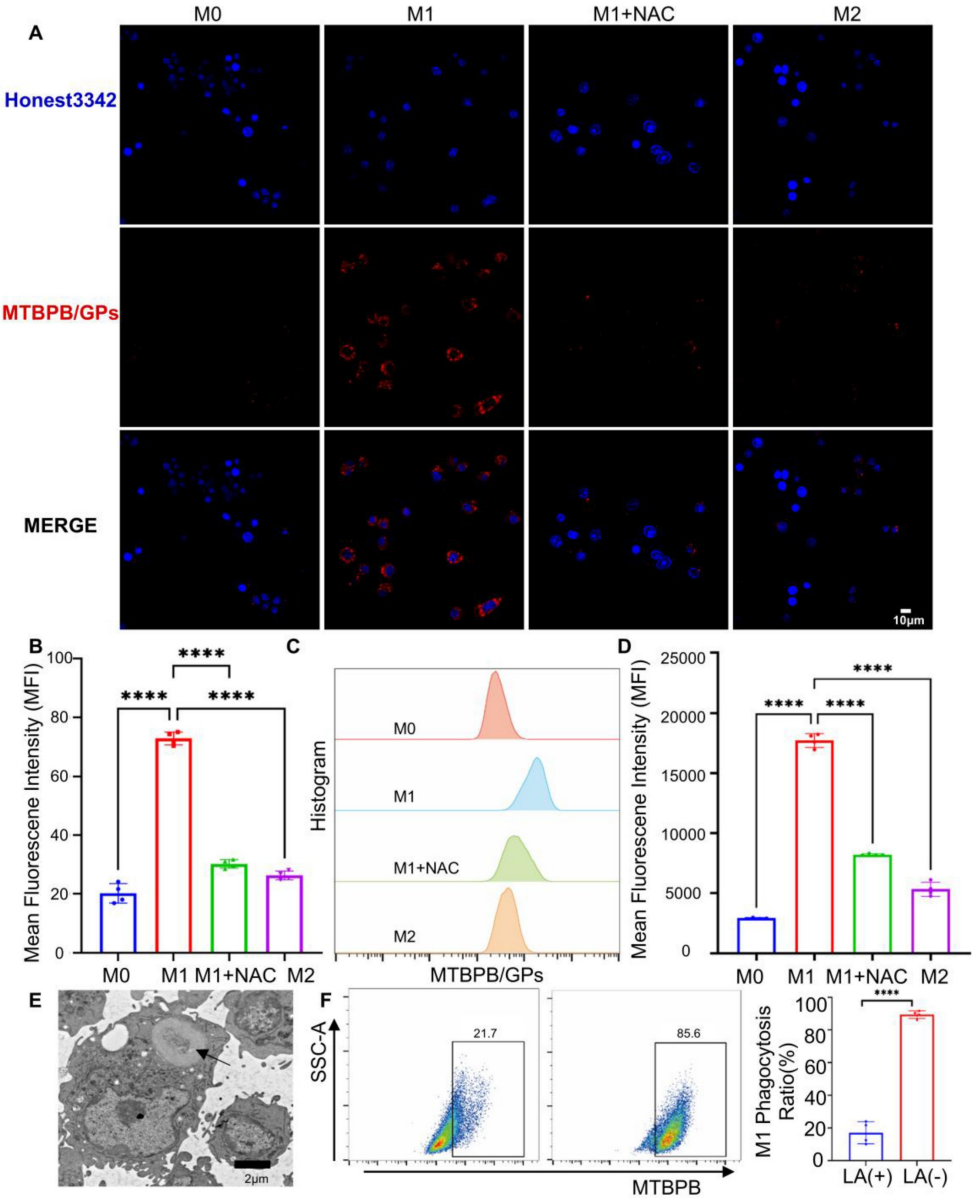
M1 macrophages activate **MTBPB/GPs** fluorescent signals. **(A)** Confocal representative images of **MTBPB/GPs** in M0, M1, M2, and M1+NAC macrophages. **(B)** Fluorescence quantification plots (n = 4). **(C)** Flow representative images of **MTBPB/GPs** in M0, M1, M2, and M1+NAC macrophages. **(D)** Flow quantitative map analysis of mean fluorescence intensity (n = 4). **(E)** TEM of **MTBPB/GPs** phagocytosis by macrophages. **(F)** Flow cytometry-based quantitative analysis plot of the phagocytosis of **MTBPB/GPs** by M1 macrophages, both before and after 12 hours of LA treatment (n = 4).^ ***^P < 0.001, ^****^ P < 0.0001.

Next, we evaluated the fluorescence activation of **MTBPB/GPs** in different macrophage subtypes. Confocal imaging revealed that M1 macrophages exhibited a fluorescence signal 3.6 times higher than M0 and 2.8 times higher than M2 cells, aligning with their elevated ROS levels (**Figure 3A, B**). Flow cytometry analysis further validated this trend, with M1 macrophages displaying 6.0- and 3.3-fold higher fluorescence compared to M0 and M2 cells, respectively. Notably, treatment with the ROS scavenger N-acetylcysteine (NAC) significantly attenuated the fluorescence signal in M1 macrophages, reducing it to less than 50% of the original level (**Figure 3C, D**). These findings confirm that fluorescence activation of **MTBPB/GPs** is tightly correlated with intracellular oxidative stress and is selectively triggered by the ROS-enriched environment of M1 macrophages. Mechanistically, the boronate ester moiety on **MTBPB** is cleaved by H₂O₂, which is abundantly produced in M1 macrophages upon stimulation with LPS and IFN-γ, leading to fluorescence activation. In contrast, M0 macrophages under homeostatic conditions exhibit low ROS levels, insufficient to activate the probe.

Previous studies have shown that GPs are internalized by macrophages via the dectin-1 receptor-mediated endocytic pathway 39. Transmission electron microscopy confirmed that **MTBPB/GPs** were successfully phagocytosed by macrophages (**Figure 3E**). To determine whether this mechanism applies to M1 macrophages, we pretreated cells with laminin (LA), a known dectin-1 receptor inhibitor. LA treatment reduced the uptake of **MTBPB/GPs** to approximately 20% of the control group, highlighting the pivotal role of dectin-1-mediated endocytosis in probe internalization (**Figure 3F**).

To investigate the subcellular localization of the probe, we performed colocalization analysis of **MTBPB/GPs** and lysosomes in LPS-stimulated M1 macrophages using confocal microscopy. The Manders' overlap coefficient was calculated to be 0.58, indicating moderate colocalization (**Figure S17**). These results suggest that while part of the probe localizes to acidic lysosomes, a substantial fraction escapes into the cytosol, where the pH is more favorable for H₂O₂-mediated activation. Moreover, during M1 polarization, cytosolic H₂O₂-primarily derived from NADPH oxidase and iNOS signaling-plays a central role in mediating oxidative stress and thus enables efficient intracellular activation of **MTBPB/GPs** 55, 62.

### Biocompatibility evaluation of MTBPB/GPs

The biosafety of **MTBPB/GPs** is essential for their translational potential in *in vivo* applications. We first evaluated the cytocompatibility of **MTBPB/GPs** using RAW264.7 macrophages. Cells were incubated with varying concentrations of **MTBPB/GPs** for 24 h, and viability remained above 90% across all tested doses, indicating minimal cytotoxicity (**Figure S18A**). Moreover, the phagocytosis of **MTBPB/GPs** did not impair macrophage migration, further supporting their cellular biocompatibility (**Figure S19**). To assess potential cytotoxic effects on non-immune cells, primary cardiomyocytes, hepatocytes, and renal tubular epithelial cells were treated with **MTBPB/GPs** under the same conditions. All tested cell types exhibited high viability (> 90%), suggesting favorable biosafety across major organ-derived cells (**Figure S18B-D**).

The *in vivo* biosafety profile of **MTBPB/GPs** was further assessed in C57BL/6 mice following daily oral administration for seven consecutive days. Hematological analysis showed no significant changes in leukocyte, erythrocyte, hemoglobin, or differential blood counts compared with untreated controls (**Figure S20**). Liver and kidney function markers, including alanine aminotransferase (ALT), aspartate aminotransferase (AST), blood urea nitrogen (BUN), and creatinine (CR), remained within normal ranges (**Figure S21A, B**), suggesting weak hepatotoxicity or nephrotoxicity. Histological examination of the heart, liver, spleen, lungs, and kidneys was performed at days 1, 3, and 7 after daily oral administration of a high dose (100 mg/kg) of **MTBPB/GPs**. Hematoxylin and eosin (H&E) staining revealed no evident tissue damage, inflammatory infiltration, or pathological abnormalities in any of the major organs (**Figure S22**), indicating excellent systemic biocompatibility. Given the exogenous nature of **MTBPB/GPs**, their immunogenicity was also assessed. No apparent systemic immune activation was observed following repeated oral administration, as evidenced by stable levels of inflammatory cytokines (**Figure S23A, B**). Collectively, these results demonstrate that **MTBPB/GPs** possess excellent cytocompatibility, systemic biosafety, and low immunogenicity, supporting their suitability for oral administration and translational application *in vivo*.

### Oral MTBPB/GPs for monitoring transplant rejection

**Figure 4:**
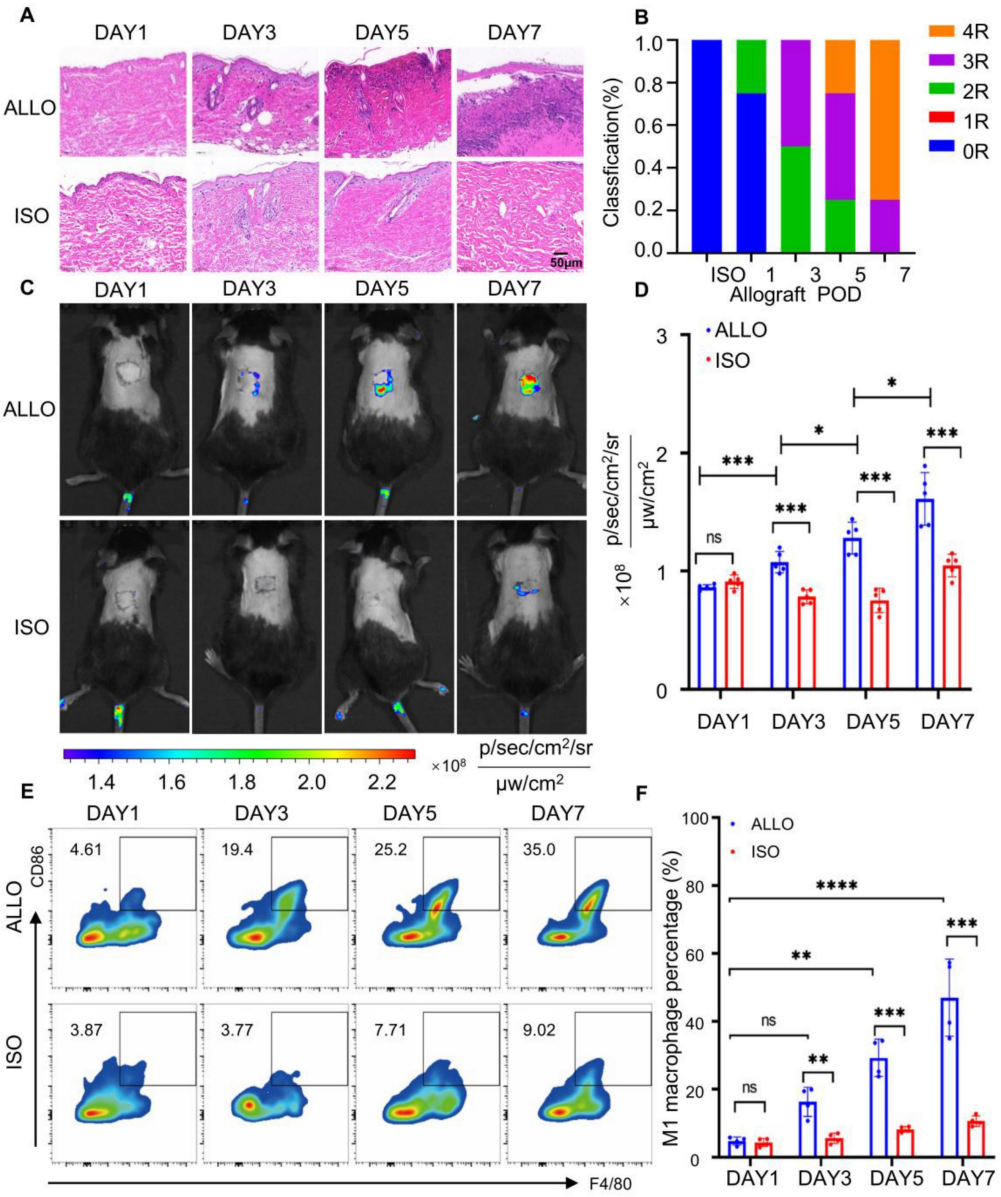
Establishment of a skin graft rejection model and monitoring of graft rejection using oral administration of **MTBPB/GPs. (A)** Histologic analysis of grafted skin at POD1, 3, 5, and 7 (n = 4). **(B)** Grade of rejection of allografts and isografts assessed by Banff classification. **(C)** Representative images of oral **MTBPB/GPs** at different time points after transplantation (POD1, 3, 5, and 7). **(D)** Fluorescence quantification image of the transplanted skin (n = 5). **(E)** Representative flow cytometry contour plots show F4/80^⁺^ CD86^⁺^ double-positive M1 macrophage populations in graft tissues. **(F)** Quantification of flow cytometry. ^*^P < 0.05, ^***^P < 0.001, ^****^ P < 0.0001.

A murine skin transplantation model was established to evaluate allograft rejection and probe performance. C57BL/6 mice served as recipients, with donor skin obtained from either syngeneic C57BL/6 (isograft) or allogeneic BALB/c mice (allograft). In the isograft group, the transplanted skin remained structurally intact and gradually integrated with the host tissue. In contrast, the allograft displayed progressive wrinkling and eventual detachment (**Figure S24**). Histopathological evaluation was conducted based on the Banff classification system 63, 64. On postoperative day 1 (POD1), both groups exhibited a rejection grade of 0R, characterized by intact epidermal and dermal architecture with minimal inflammatory infiltration. By POD7, the allograft skin showed massive leukocyte infiltration and extensive epidermal and dermal necrosis, reaching a rejection grade of ≥ 3R (**Figure 4A, B**). In comparison, the isograft maintained normal architecture with only mild inflammatory changes. These results confirm the occurrence and progression of acute rejection in the allograft group.

To monitor probe distribution and activation, *in vivo* fluorescence imaging was performed at 0, 6, 12, 24, 36, and 48 h following oral administration of **MTBPB/GPs** (**Figure S25A, B**). Fluorescence intensity at the graft site peaked at 36 h, which was subsequently selected as the optimal imaging time point. Quantitative analysis revealed that the fluorescence signal in allograft skin on POD7 was 1.87-fold higher than on POD1. Notably, fluorescence in the allograft group began to increase as early as POD3, reaching a 1.54-fold elevation compared to the isograft by POD7 (**Figure 4C, D**), indicating probe activation in response to progressive rejection (**Figure S26**).

To evaluate the biodistribution of **MTBPB/GPs**, ex vivo fluorescence imaging of major organs (heart, liver, spleen, lungs, kidneys) was performed at 24 and 36 h post-administration on POD6. Strong fluorescence signals were observed in the liver and kidneys (**Figure S27A-D**), consistent with previous reports indicating that these are the primary clearance organs for orally administered GPs 65-67.

To confirm that the fluorescence signals originated from probe activation by pro-inflammatory M1 macrophages, we performed immunofluorescence staining of graft sections using CD68 and iNOS as markers for M1 macrophages 59, 60. Immuno-staining on POD1, 3, 5, and 7 revealed progressively increasing M1 macrophage infiltration in the allograft, which became significantly elevated from POD3 onward. By POD7, the number of CD68^⁺^ iNOS^⁺^ cells in the allograft was 9.24-fold higher than in the isograft group (**Figure S28**). These results were further validated by flow cytometry, which showed a 2.9-fold increase in M1 macrophages at POD3 and a 4.2-fold increase by POD7 (**Figure 4E, F**). Collectively, these data demonstrate that **MTBPB/GPs** preferentially accumulate and activate at sites of allograft rejection, where M1 macrophages are enriched. The observed correlation between fluorescence intensity and M1 macrophage infiltration confirms the utility of **MTBPB/GPs** for noninvasive, real-time monitoring and early diagnosis of transplant rejection.

### Oral MTBPB/GPs to monitor immunosuppressant efficacy

**Figure 5:**
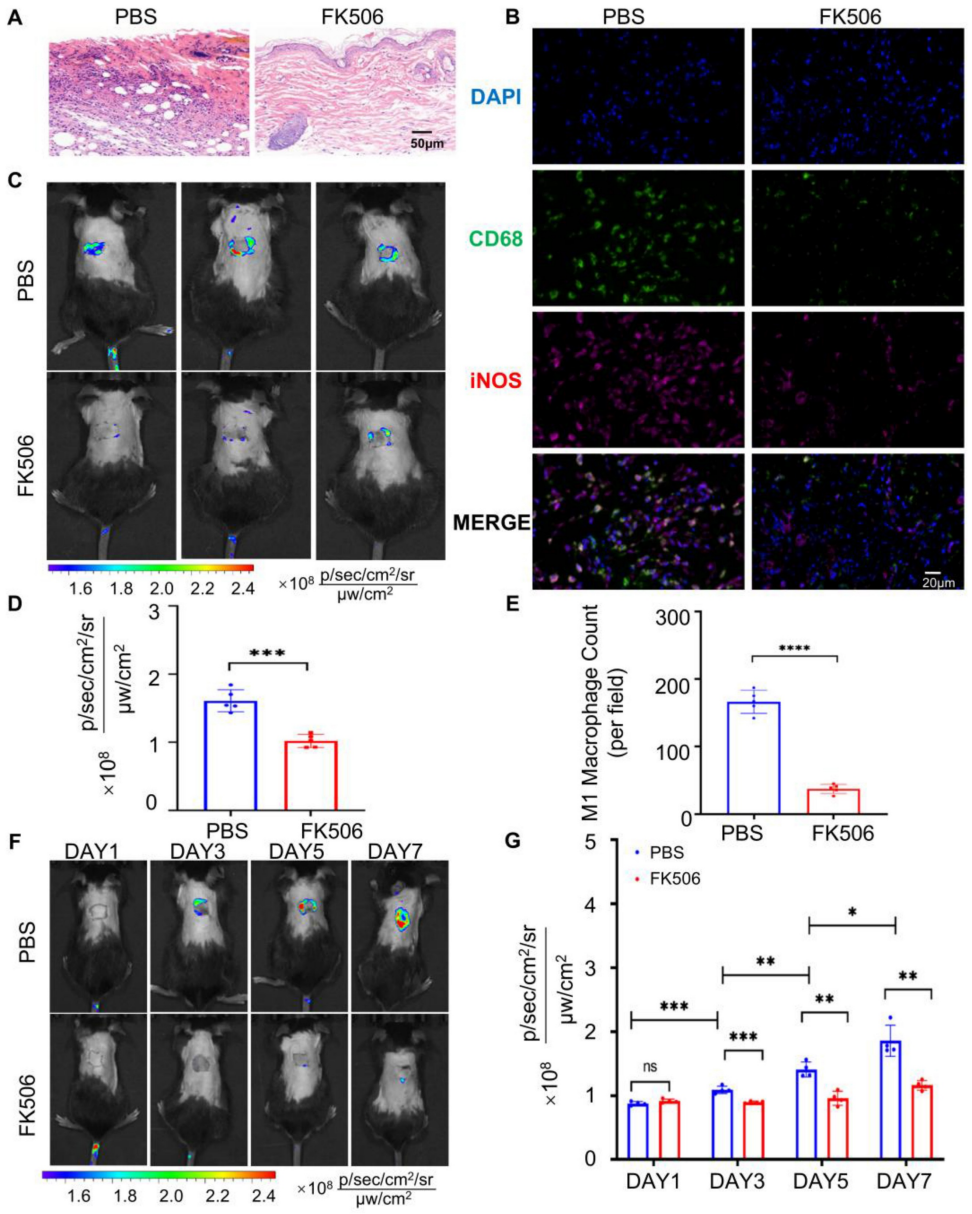
**MTBPB/GPs** were administered orally to assess the effectiveness of the immunosuppressive therapy. **(A)** Pathology results of transplanted skin after 7 days of subcutaneous injection of FK506 or PBS (n = 4). **(B)** Immunofluorescence staining of CD68^⁺^ iNOS^⁺^ M1 macrophages in allografted skin after 7 days of subcutaneous injection of FK506 or PBS; FITC-labeled for staining CD68, Cy3-labeled for staining iNOS; nuclei were stained with DAPI. **(C)** Whole-body fluorescence imaging was conducted on FK506- or PBS-treated allograft mice over a period of 7 consecutive days. **(D)** Quantification of the transplanted skin (n = 5). **(E)** Quantification of CD68^+^ iNOS^+^ M1 macrophages in transplanted skin (n = 4). **(F)** Whole-body fluorescence imaging was carried out on allograft subcutaneous injection of FK506 or PBS over a span of 7 consecutive days (POD 1, 3, 5, and 7). **(G)** Quantification of transplanted skin (n = 4). ^*^P < 0.05, ^**^P < 0.01, ^***^P < 0.001, ^****^ P < 0.0001.

Real-time assessment of immunotherapy efficacy is essential for managing transplant rejection and minimizing post-transplant complications. To this end, we investigated the utility of orally administered **MTBPB/GPs** for monitoring the effectiveness of immunosuppressive treatments. Tacrolimus (FK506), a first-line immunosuppressant, primarily inhibits the calcineurin pathway in T cell, thereby suppressing interleukin-2 production and T-cell proliferation 68, 69. Additionally, FK506 effectively inhibits macrophage proliferation and the secretion of TNF-α 70, 71. We established an allograft rejection model in mice and administered FK506 subcutaneously at a dosage of 3 mg/kg daily for 7 days. Histopathological analysis of grafted skin on POD 7 revealed that the FK506-treated group maintained intact skin architecture, whereas the control group exhibited extensive necrosis and significant inflammatory cell infiltration (**Figure 5A**). Immunofluorescence analysis quantified CD68^⁺^ iNOS^⁺^ M1 macrophages within the grafted skin, showing that the control group had approximately 4.4 times more M1 macrophages than the FK506-treated group **(Figure 5B** and** 5E**). This indicates that FK506 effectively reduces M1 macrophage infiltration into the graft.

Subsequently, we investigated whether oral administration of **MTBPB/GPs** could facilitate imaging of M1 macrophages within the graft, thereby enabling evaluation of therapeutic efficacy. After 7 days of FK506 treatment, the fluorescence signal decreased to approximately half of the pre-treatment level (**Figure 5C, D**), suggesting that oral **MTBPB/GPs** can reflect the efficacy of FK506. To assess the potential of **MTBPB/GPs** for monitoring immunosuppressant efficacy, we orally administered **MTBPB/GPs** on the day after surgery and conducted imaging on POD 1, 3, 5, and 7 (**Figure S29**). In the FK506-treated group, fluorescence intensity began to decrease on POD3 and was reduced by 1.6-fold compared to the control group by POD7 (**Figure 5F, G**). These findings demonstrate that **MTBPB/GPs** can monitor M1 macrophage dynamics over time, offering a non-invasive approach for real-time evaluation of immunosuppressive therapy efficacy.

### Mechanism of oral MTBPB/GPs targeting graft tissue

Following oral administration, GPs are internalized by intestinal macrophages via M cells in Peyer's patches, after which they migrate through the lymphatic system to peripheral tissues 72, 73. This macrophage-mediated delivery route is critical for enabling specific probe accumulation at inflammatory sites 74, 75. To evaluate the necessity of macrophage targeting for effective graft localization, we compared the biodistribution of free **MTBPB** versus **MTBPB/GPs**. As shown in **Figure S30A-C**, free **MTBPB** failed to accumulate at the graft, while **MTBPB/GPs** exhibited a 1.67-fold higher fluorescence signal at the transplant site, confirming the pivotal role of GPs in enabling efficient macrophage-mediated delivery.

**Figure 6:**
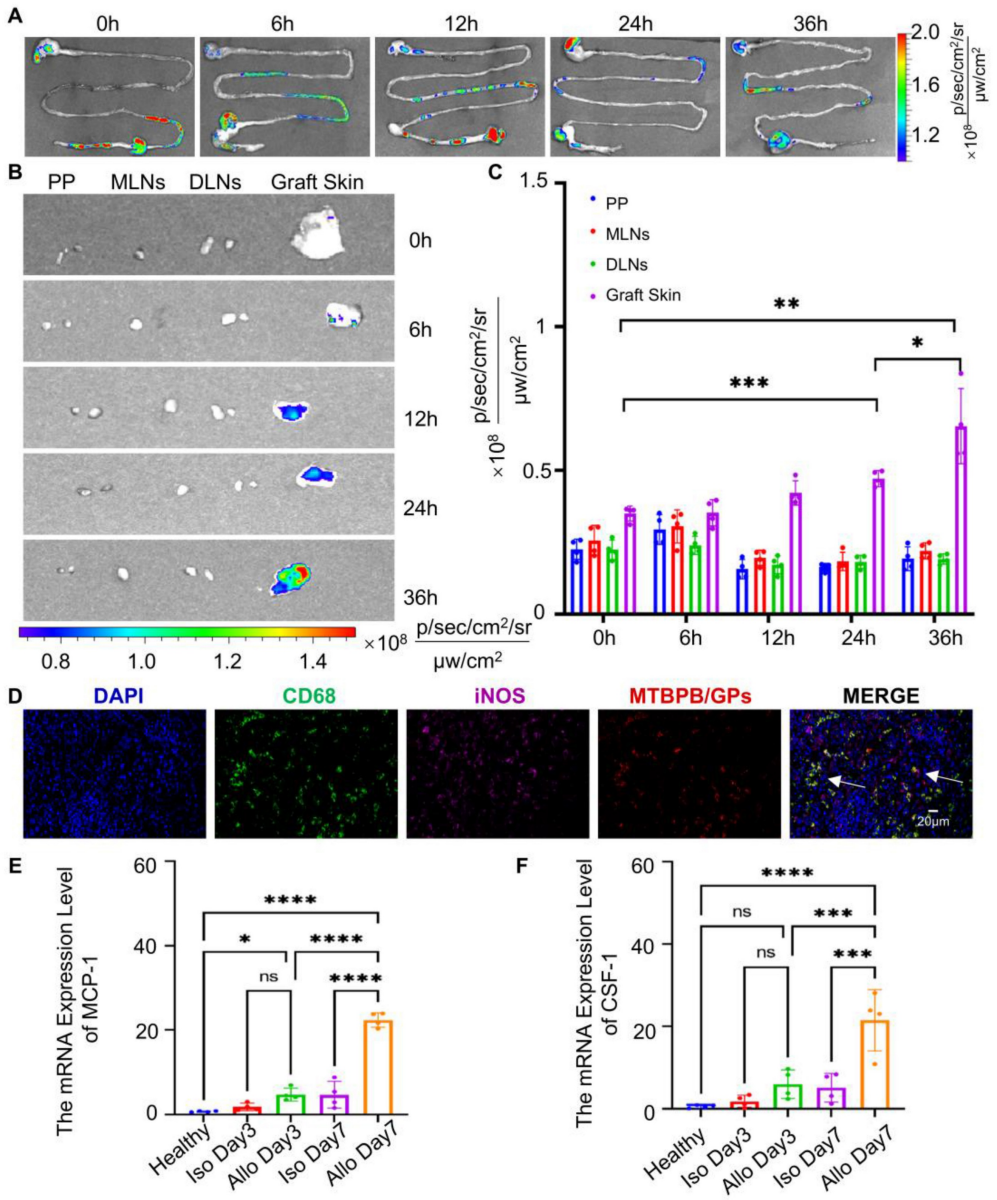
*In vivo* transport of **MTBPB/GPs** to grafted skin. **(A)** Distribution of **MTBPB/GPs** in the ex vivo gastrointestinal tract at various time intervals (0, 6, 12, 24, 36 h) of allografted mice on POD 6 (n = 4). **(B)** Representative images of **MTBPB/GPs** in lymph nodes (PP, MLNs, DLNs) and graft skin and **(C)** quantitative fluorescence intensity analysis graph (n = 4). **(D)** Immunofluorescence staining of allografted skin on POD6 after oral administration of **MTBPB/GPs** for 36 h. DAPI stained nuclei, FITC labeled CD68, Cy5 labeled iNOS, **MTBPB/GPs** autofluoresced in red. **(E)** expression levels of MCP-1 and **(F)** CSF-1 in both healthy and transplanted skin (n = 4). ^*^P < 0.05, ^**^P < 0.01, ^***^P < 0.001, ^****^ P < 0.0001.

In our previous work 39, we demonstrated that orally administered GPs are taken up by M cells in Peyer's patches and phagocytosed by intestinal macrophages. These macrophages then migrate through gut-associated lymphoid tissues (GALT), including the mesenteric lymph nodes (MLNs) and draining lymph nodes (DLNs), and ultimately home to sites of inflammation. This process was confirmed by time-resolved fluorescence imaging, showing sequential probe accumulation in Peyer's patches, MLNs, DLNs, and the graft. In this study, we employed the same GP-based delivery platform for the H₂O₂-activatable AIE probe **MTBPB**. First, we performed ex vivo fluorescence imaging of the gastrointestinal tract at 0, 6, 12, 24, and 36 h post-oral administration. Across all time points, the GI tract exhibited minimal fluorescence signal (**Figure 6A**), suggesting that **MTBPB/GPs** remain in an “off” state in the gastrointestinal environment, likely due to the lack of sufficient ROS, thus preserving imaging specificity.

To further elucidate the spatiotemporal behavior of the probe, we conducted a time-course analysis (0-36 h) using ex vivo organ imaging and fluorescence histology. Fluorescence signals in lymphoid tissues remained low, consistent with probe quiescence during systemic trafficking (**Figure S31**). In contrast, graft-specific fluorescence progressively increased over time, indicating selective probe activation within the inflammatory microenvironment enriched in H₂O₂ due to M1 macrophage infiltration (**Figure 6B, C**). Immunofluorescence analysis on POD7 confirmed significant colocalization between **MTBPB/GPs** and CD68^⁺^ iNOS^⁺^ M1 macrophages in the allograft (**Figure 6D**). These findings support a mechanism in which intestinal macrophages engulf **MTBPB/GPs** and migrate via the lymphatic system to the graft, where they undergo M1 polarization in response to inflammatory stimuli, thereby generating elevated ROS levels that activate the probe.

During graft rejection, immune cells particularly T cells and macrophages—secrete chemokines that guide monocyte and macrophage recruitment to the graft 76-78. Monocyte chemoattractant protein-1 (MCP-1) and colony-stimulating factor-1 (CSF-1) are key mediators of this chemotactic process 79, 80. To investigate their involvement, we quantified mRNA expression levels of MCP-1 and CSF-1 in graft tissues at POD3 and POD7. Both chemokines were significantly upregulated by POD7 (**Figure 6E, F**), consistent with increased macrophage recruitment and M1 polarization at later stages of rejection. These findings confirm the mechanism of macrophage-mediated trafficking of **MTBPB/GPs** following oral administration. The probe remains inactive during transit and is selectively activated *in situ* by oxidative stress from M1 macrophages at the site of rejection. This spatially restricted activation provides high imaging specificity and supports the clinical utility of **MTBPB/GPs** as a noninvasive platform for early detection and monitoring of transplant rejection.

## Discussion

Biopsy remains the clinical gold standard for diagnosing transplant rejection 6, yet it is invasive and carries risks such as bleeding, infection, and sampling error. In routine practice, non-invasive alternatives rely on serum biomarkers or imaging assessments 3, 4, 8. However, commonly used biomarkers like creatinine and urea suffer from poor specificity, as their elevations may reflect various non-rejection-related causes of graft dysfunction 81, 82. Similarly, conventional imaging modalities such as ultrasound and MRI provide only anatomical or perfusion-level information and lack sensitivity for early immune activation or real-time tracking of immune cell infiltration.

To overcome these limitations, we developed **MTBPB/GPs** an orally administered, H₂O₂-activatable, AIE-based biomimetic probe designed for macrophage-targeted imaging of transplant rejection. This system offers three key advantages: (i) excellent biosafety, as demonstrated by both *in vitro* and *in vivo* evaluations; (ii) high sensitivity enabled by selective fluorescence activation in M1 macrophage-rich, oxidative microenvironments; and (iii) the capability to dynamically monitor immune response and treatment efficacy with a single oral dose.

Despite these advantages, the current imaging system is limited by shallow penetration depth, restricting its utility in monitoring deeply situated organs such as kidney transplants. To address this, future iterations of the probe will be designed to operate within the second near-infrared window (NIR-II), which offers deeper tissue penetration and improved spatial resolution. In addition, combining this probe with other imaging modalities-such as PET, CT, or MRI-could enable a multimodal imaging strategy, thereby improving diagnostic accuracy and spatiotemporal resolution. In conclusion, **MTBPB/GPs** complement and extend current diagnostic strategies by providing a non-invasive, macrophage-responsive, and dynamically activatable platform for early detection and longitudinal monitoring of transplant rejection. This approach may significantly advance personalized transplant management and improve patient outcomes.

## Conclusion

In this study, we developed an innovative platform for non-invasive immune monitoring of transplant rejection using an H₂O₂-activatable biomimetic probe based on aggregation-induced emission (AIE) glucan particles (**MTBPB/GPs**). The probe demonstrates excellent biocompatibility, characterized by low cytotoxicity, minimal hemolytic activity, negligible immunogenicity, and low systemic toxicity, making it a promising alternative to conventional biopsy for graft evaluation. Importantly, **MTBPB/GPs** enable selective fluorescence activation by pro-inflammatory M1 macrophages, allowing for early and highly sensitive detection of transplant rejection. A single oral administration facilitates dynamic tracking of the immune response and therapeutic efficacy of immunosuppressive regimens, enabling real-time monitoring without repeated interventions. By integrating inflammation-triggered activation with macrophage-mediated delivery, **MTBPB/GPs** offer a high signal-to-noise ratio, excellent specificity, and operational simplicity. Collectively, this approach holds strong translational potential to enhance immune surveillance, reduce dependence on invasive procedures, and support personalized post-transplant management.

## Supplementary Material

Supplementary figures and tables.

## Abbreviations

ALT: alanine aminotransferase
AST: aspartate aminotransferase
AIE: aggregation-induced emission
BUN: blood urea nitrogen
CR: creatinine
CSF-1: colony-.stimulating factor-1
DLNs: draining lymph nodes
FI-IR: Fourier Transform Infrared
FK506: Tacrolimus
^18^F-FDG: ^18^F-fluorodeoxyglucose
GPs: glucan particles
H₂O₂: hydrogen peroxide
HPLC: high-performance liquid chromatography
ICT: intramolecular charge transfer
ISC: intersystem crossing
LPS: lipopolysaccharide
LA: laminin
MLNs: mesenteric lymph nodes
MCP-1: Monocyte chemoattractant protein-1
MRI: magnetic resonance imaging
NIR-II: second near-infrared window
POD: postoperative day
PP: Peyer's patches
PET: positron emission computed tomography
ROS: reactive oxygen species
SOC: spin-orbit coupling
SPIONs: superparamagnetic iron oxide nanoparticles
SEM: scanning electron microscopy
TR: transplant rejection
TEM: transmission electron microscopy
XRD: X-ray diffraction
YM: yeast microcapsules
