# Supplementary material for: Activatable biomimetic probe with aggregation-induced emission characteristics for non-invasive monitoring of allograft rejection
Source: Theranostics. 2025 May 30;15(13):6572–92. doi: 10.7150/thno.110866 (PMC12160014; doi:10.7150/thno.110866)
Supplement: Supplementary file 1 — Supplementary figures and tables. [file thnov15p6572s1.pdf]

Activatable biomimetic probe with aggregation-induced emission characteristics for non-invasive monitoring of allograft rejection

Mengdan Ding[#1,2,3], Fang He[#1,2,3], Shuangze Han[1,2,3], Wuqi Zhou[1,2,3], Tian Huang[1,2,3], Nan Cui[1,2,3], Yuanting Quan[1,2,3], Wenqu Li[1,2,3], WenyuanWang[1,2,3], Tang Gao[*1,2,3], Mingxing Xie[*1,2,3], Li Zhang[*1,2,3]

1 Department of Ultrasound Medicine, Union Hospital, Tongji Medical College, Huazhong University of Science and Technology, Wuhan 430022, China;

2 Hubei Province Clinical Research Center for Medical Imaging, Wuhan 430022, China;

3 Hubei Province Key Laboratory of Molecular Imaging, Wuhan 430022, China

[#] These authors contributed equally to this work

Corresponding author E-mail: gaotang@hust.edu.cn; xiemx@hust.edu.cn; zli429@hust.edu.cn.

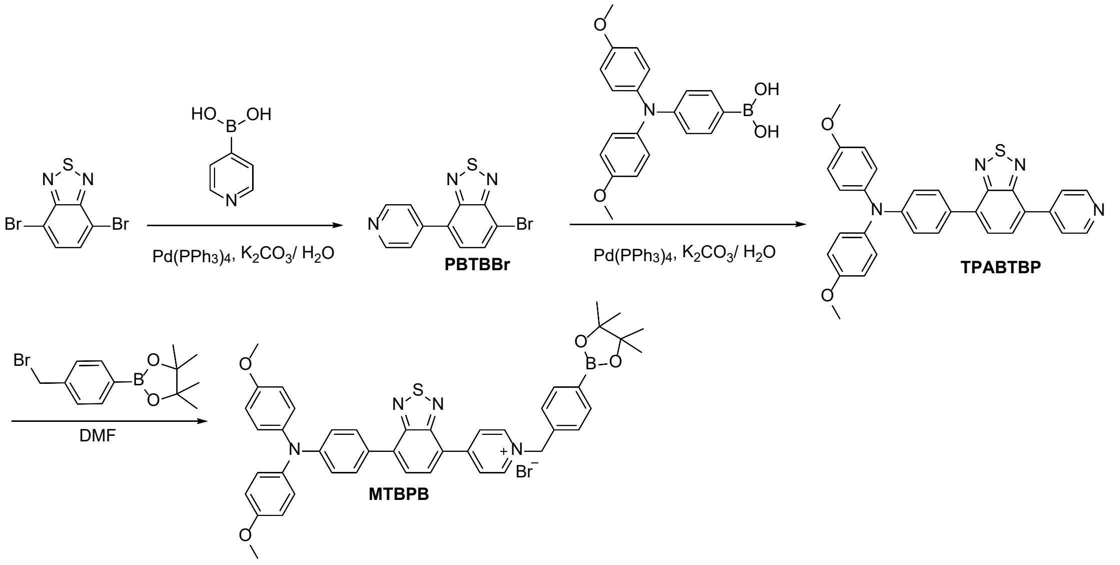

Scheme S1. Synthetic route of **MTBPB**

Synthesis of compound ***PBTBBr:*** Pyridine-4-boronic acid (200 mg, 1.62 mmol), 4,7-dibromo-benzo[c][1,2,5]thiadiazole (472.8 mg, 1.62 mmol), potassium carbonate (250 mg), and Pd(PPh3)4 (20 mg) were added to a 100 mL round-bottom flask fitted with a condenser. The mixture was dissolved in 10 mL of DMF and 2 mL of water under a nitrogen atmosphere, forming a solution that was stirred at 400 rpm and heated to reflux at 80°C for 24 hours. After cooling to 25°C, the reaction mixture underwent extraction with ethyl acetate (three repetitions) under anhydrous conditions. The combined organic layers were dried over anhydrous sodium sulfate. The solvent was removed under reduced pressure (0.1 MPa vacuum) to yield a crude product. Purification was carried out by silica gel column chromatography using a petroleum ether/ethyl acetate mixture (25:1, v/v) as the eluent, affording **PBTBBr** as a solid (226.2 mg, 48% yield).^{1}H NMR (600 MHz, $CDCl_3$), δ(ppm): 8.78-8.79 (m, 2H), 7.98-7.99 (d, 1H), 7.86 - 7.87(d, 2H), 7.69-7.70 (d, 1H); ^{13}CNMR (150 MHz, CDCl3), δ(ppm):153.96, 152.48, 150.22, 143.94, 132.12, 130.89, 128.92, 123.48, 115.42.

Synthesis of compound ***TPABTBP*****:** Compound **PBTBBr** (55.1 mg, 0.19 mmol) and (4-(bis(4-methoxyphenyl)amino)phenyl)boronic acid (66.3 mg, 0.19 mmol) were dissolved in 5 mL of DMF and stirred thoroughly at 600 rpm at 25°C. To this solution,

1 mL of an aqueous solution containing potassium carbonate (50 mg) was added, followed by 10 mg of Pd(PPh3)4 as a catalyst. The mixture was refluxed at 80°C under a nitrogen atmosphere for 12 hours. Upon confirmation of reaction completion via TLC, the solvent was removed under reduced pressure (0.1 MPa). The resulting product, **TPABTBP**, was purified using silica gel column chromatography with a petroleum ether/ethyl acetate mixture (25:1, v/v) as the eluent, yielding 32.8%. ^{1}H NMR (600 MHz, CDCl3), δ(ppm): 8.92- 8.93 (d, 2H), 8.58-8.57 (d, 2H), 8.07-8.09 (d, 1H), 7.88-7.90 (d, 2H), 7.83-7.85 (d, 1H), 7.16-7.17 (m, 4H), 7.05-7.06 (d, 2H), 6.88-6.90 (d, 4H), 3.83 (s, 6H); ^{13}CNMR (150 MHz, $CDCl_3$), δ(ppm): 156.63, 154.11, 153.19, 152.36, 150.13, 142.52, 139.92, 137.88, 130.87, 130.26, 127.47, 127.02, 125.83, 125.29, 124.72, 118.92, 114.91, 55.53. HRMS (ESI^+): calcd for $C_{31}H_{25}N_4O_2S^+$ $[M+H]^+$: 517.1693; found: 517.1689.

Synthesis of compound ***MTBPB*****:** A mixture of **TPABTBP** (82.6 mg, 0.16 mmol) and 4-(bromomethyl)benzene boronic pinacol ester (62.9 mg, 0.20 mmol) was dissolved in anhydrous DMF (15.0 mL) and heated to reflux for 8 hours. After the reaction mixture cooled to room temperature, the resulting precipitate was collected by filtration and washed thoroughly with acetone. A red solid was obtained with a yield of 68.5%. ^{1}HNMR (600 MHz, CD_3OD), δ(ppm): 9.05-9.07 (m, 2H), 8.96-8.97 (m, 2H), 8.40-8.42 (m, 1H), 7.96-7.99 (m, 3H), 7.85-7.86 (m, 1H), 7.73-7.74 (m, 1H), 7.50-7.54 (m, 2H), 7.10-7.12 (d, 4H), 6.91-6.97 (m, 6H), 5.85-5.87 (d, 2H), 3.80 (s, 6H), 1.20-1.34 (m, 11H), 0.88-0.99 (m, 1H); ^{13}CNMR (150 MHz, CD_3OD), δ(ppm): 158.36, 155.28, 154.87, 154.83, 154.62, 151.70, 145.48, 145.43, 141.36, 139.68, 137.80, 136.92, 135.82, 133.73, 133.71, 131.67, 129.22, 129.12, 128.59, 128.47, 127.90, 127.09, 124.56, 119.86, 117.95, 116.04, 85.52, 75.88, 64.90, 64.85, 56.01, 33.12, 30.82, 30.79, 30.64, 30.51, 30.36, 30.28, 25.22, 25.07, 23.78, 14.48. HRMS (ESI^+): calcd for $C_{44}H_{42}BN_4O_4S^+$ $[M-Br]^+$: 733.30; found: 733.3006.

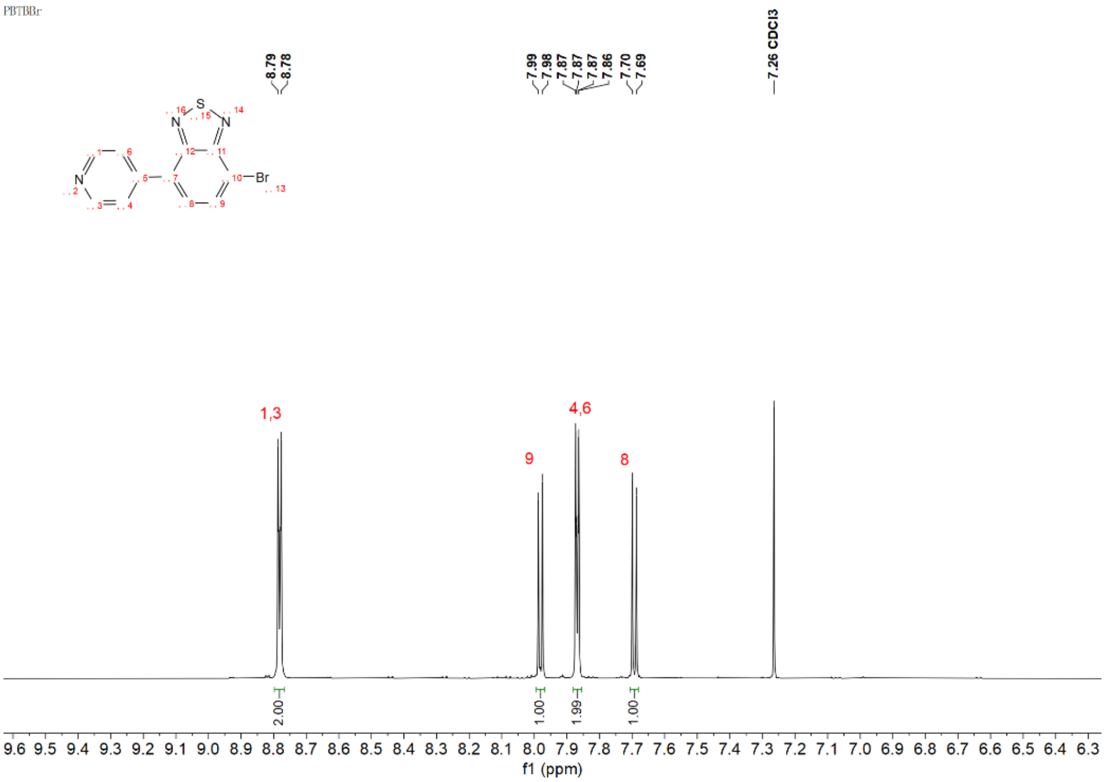

Figure S2. ^{1}HNMR spectrum of **PBTBBr**.

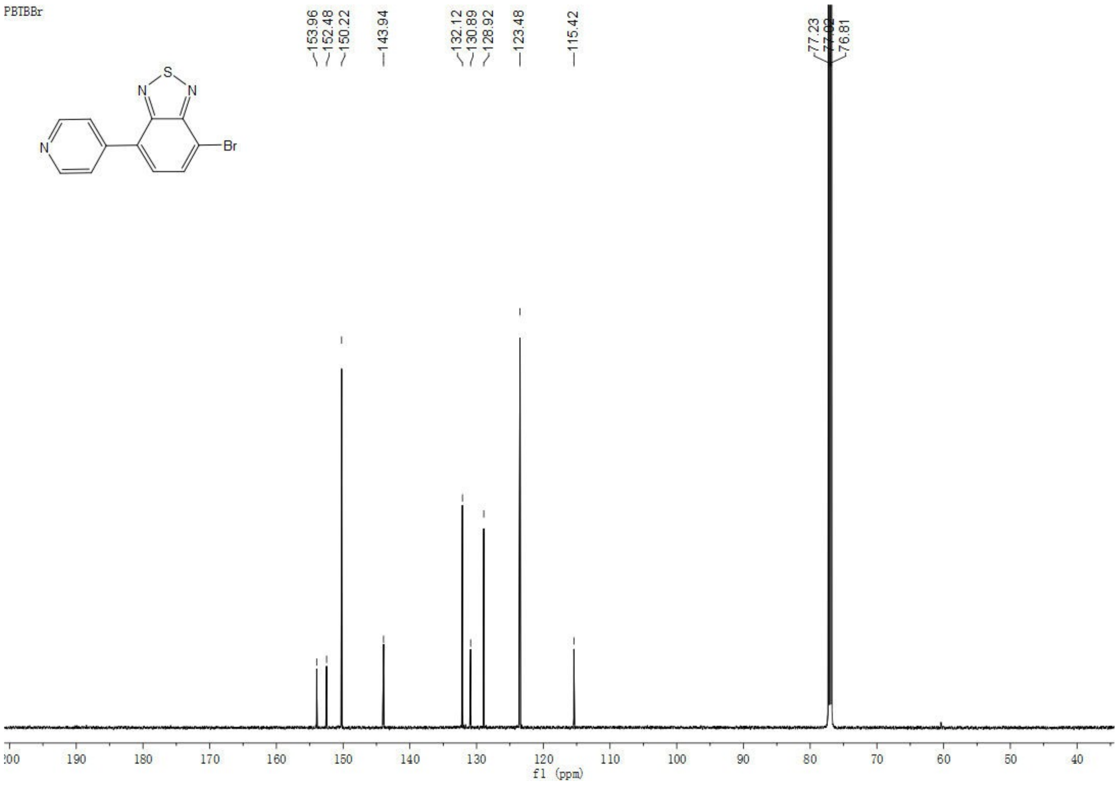

Figure S3. ^{13}CNMR spectrum of **PBTBBr**.

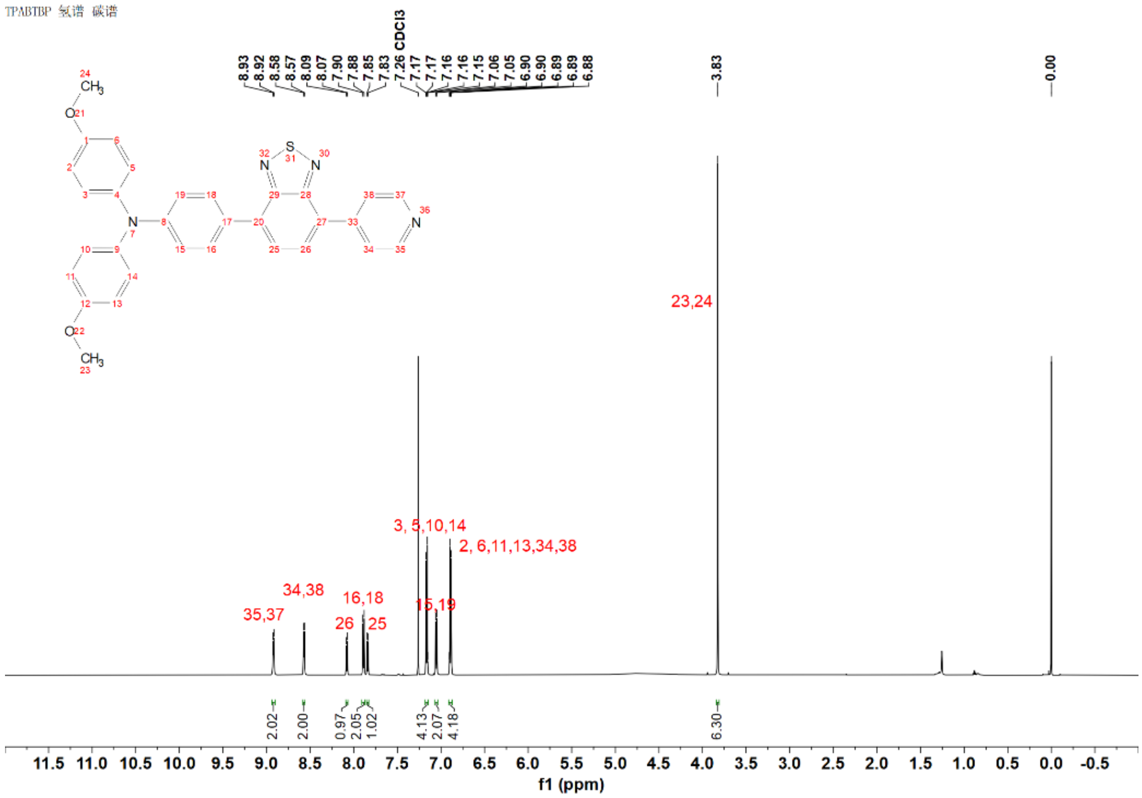

Figure S4. ^{1}HNMR spectrum of **TPABTBP.**

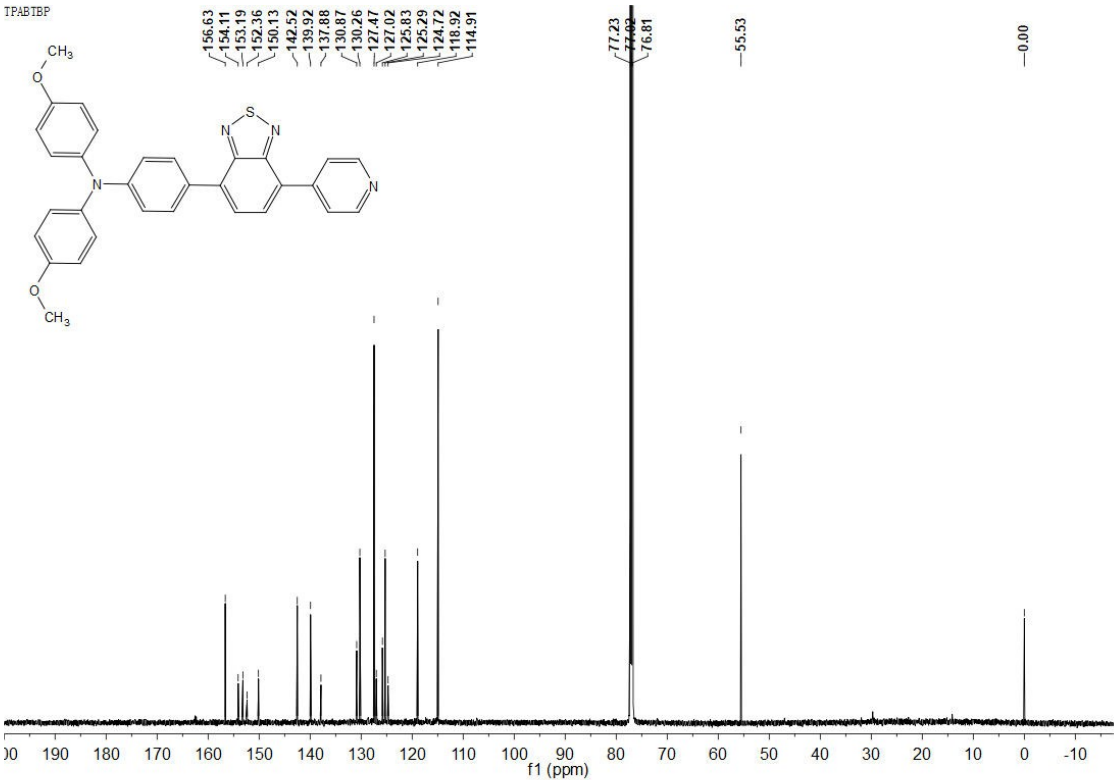

Figure S5. ^{13}CNMRspectrum of **TPABTBP.**

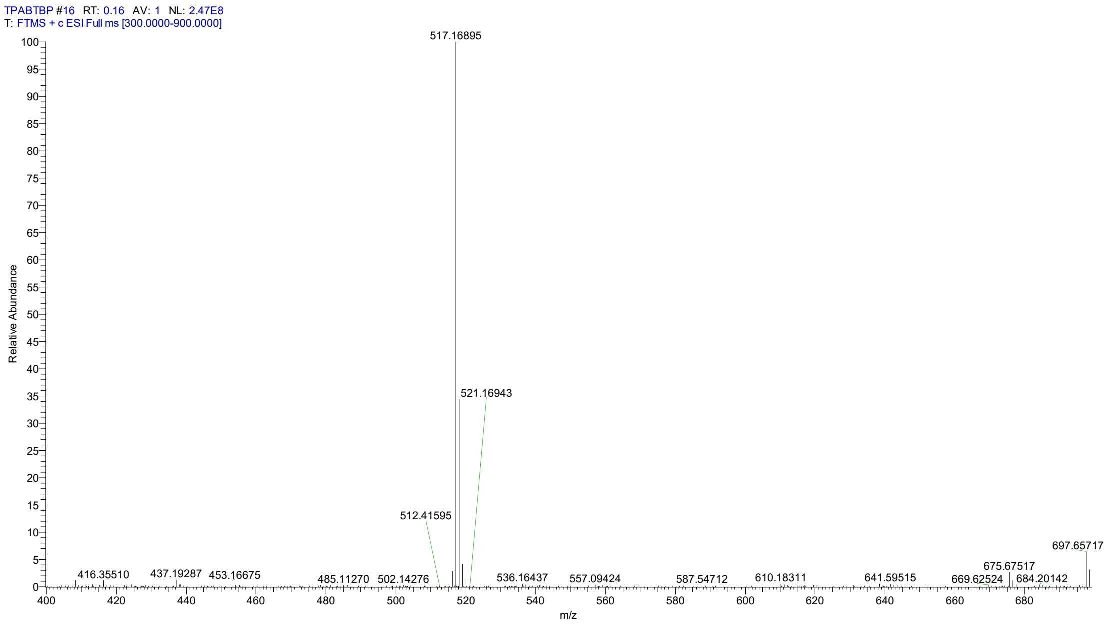

Figure S6. HRMS spectrum of **TPABTBP.**

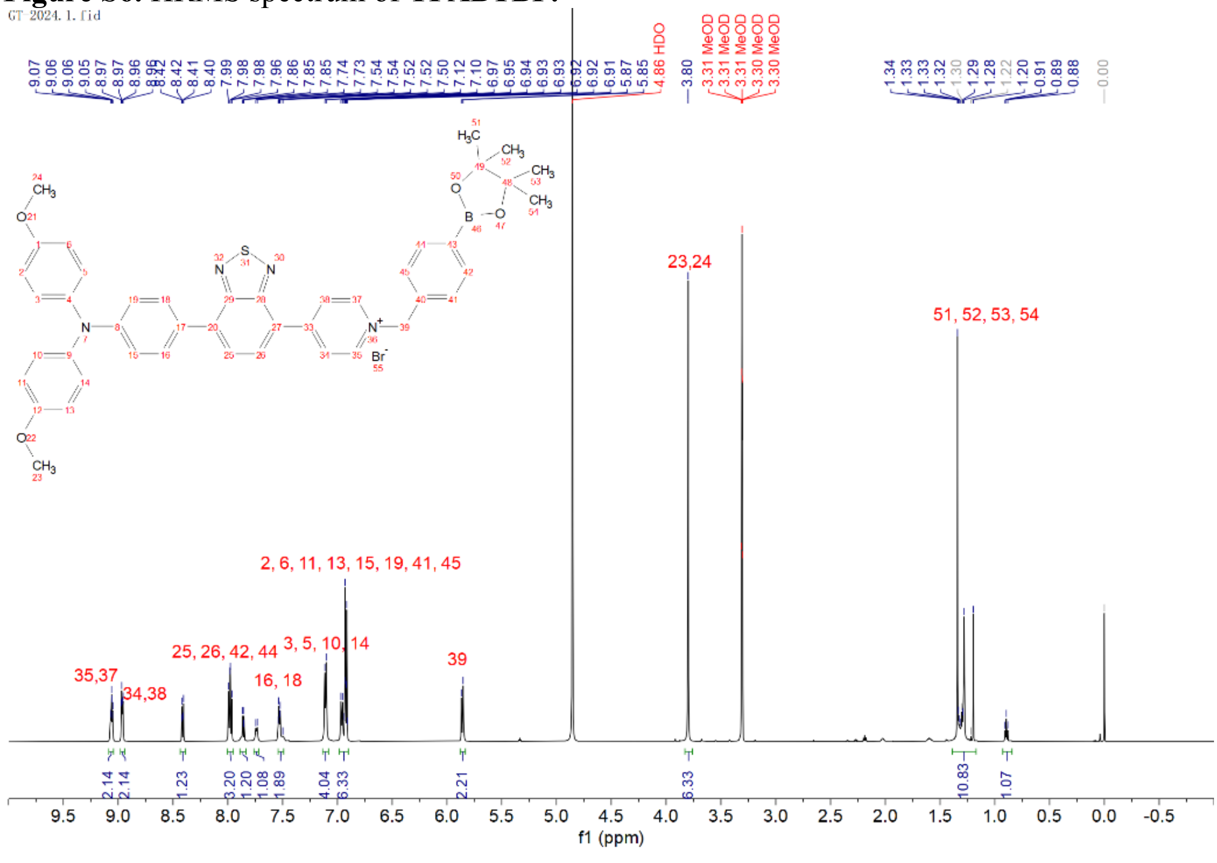

Figure S7. ^{1}HNMR spectrum of **MTBPB.**

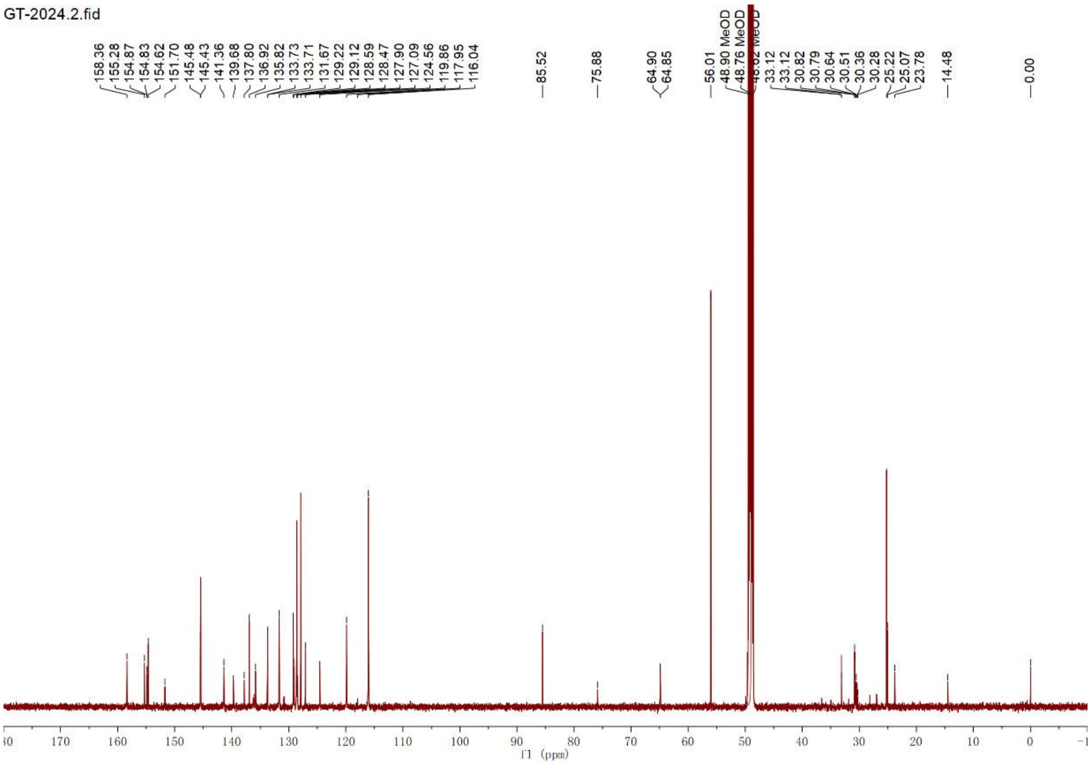

Figure S8. ^{13}CNMR spectrum of **MTBPB.**

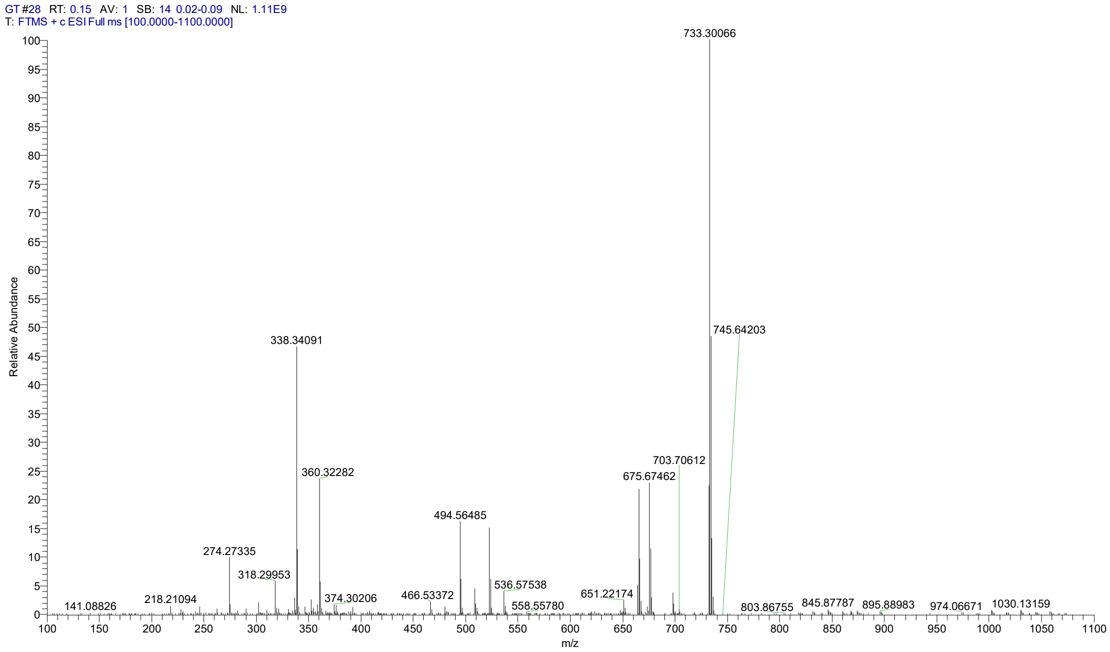

Figure S9. HRMS spectrum of **MTBPB.**

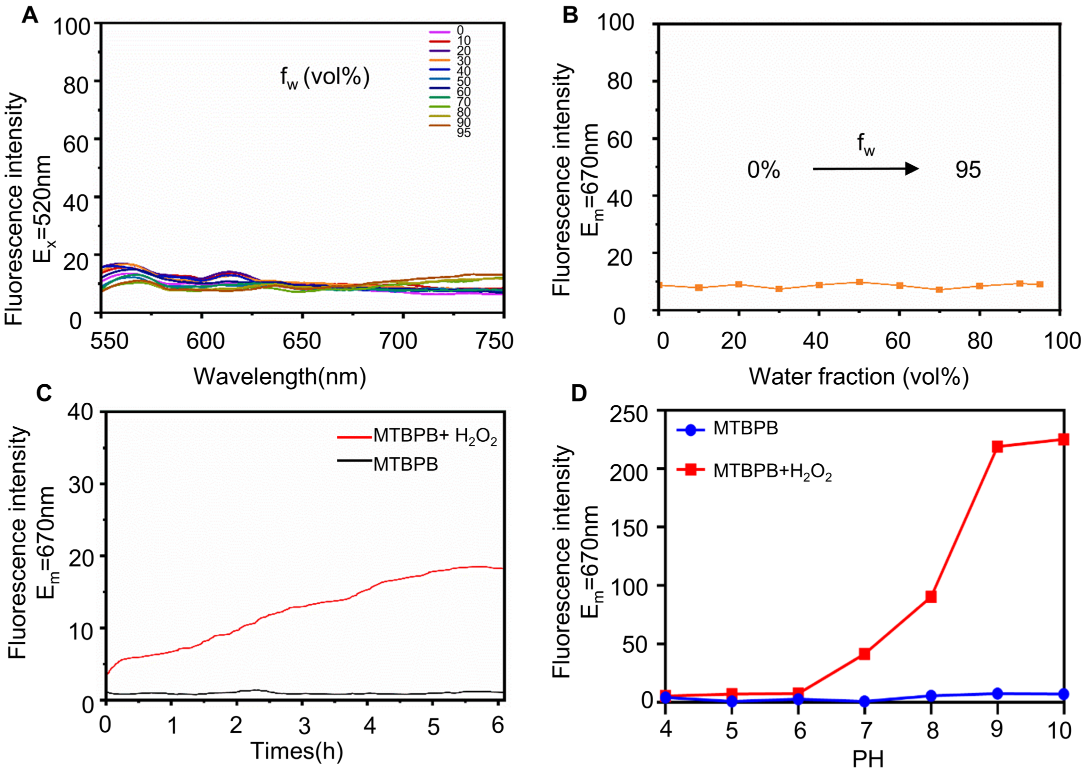

Figure S10. (A) Fluorescence emission spectra of **MTBPB** at different ratios of water/DMSO (0-95%). (B) Fluorescence intensity of **MTBPB** (10 μM) at 670 nm as a function of water/DMSO (0-95%). (C) Time-fluorescence intensity curve of **MTBPB** (10 μM) at 670 nm in the absence or presence of H_2O_2 (5 μM). (D) Fluorescence intensity of **MTBPB** (10 μM) at 670 nm in different pH (4-10) in the absence or presence of H_2O_2 (5 μM).

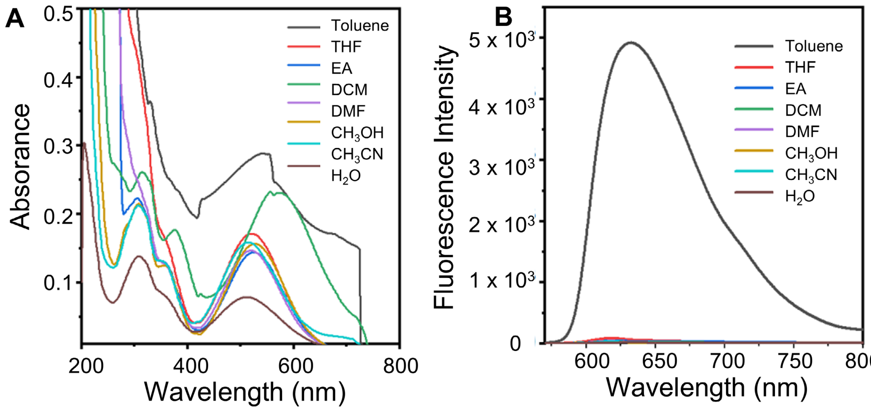

Figure S11. (A) The UV–Vis absorption spectra and (B) fluorescence emission spectra of MTBPB (10 μM) in various solvents, including toluene, tetrahydrofuran (THF), ethyl acetate (EA), dichloromethane (DCM), N,N-dimethylformamide (DMF), acetonitrile (CH_3CN), methanol (CH_3OH), and water (H_2O). MTBPB exhibits strong emission in non-polar solvent (toluene) and nearly quenched fluorescence in polar or protic solvents.

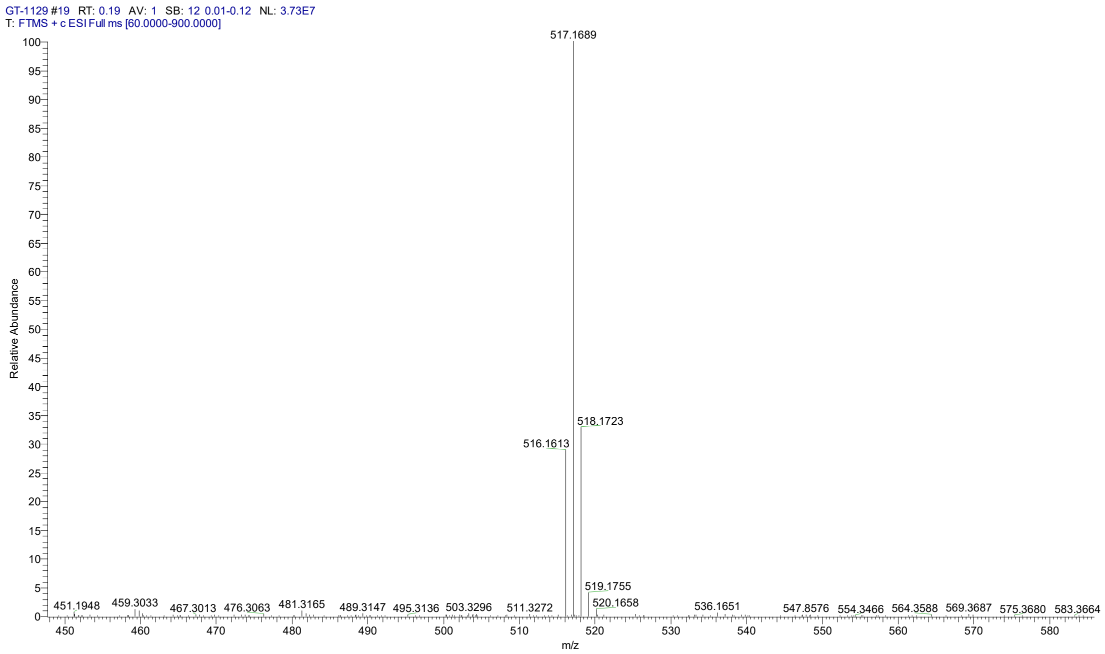

Figure S12. HRMS spectrum of **MTBPB** in the presence of H_2O_2

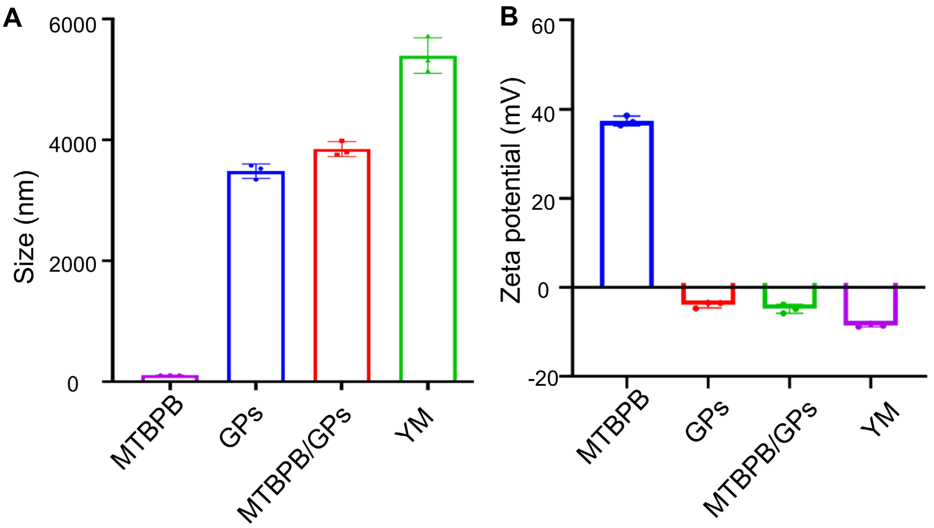

Figure S13. The (A) particle size and (B) zeta potential of **MTBPB**, GPs, **MTBPB/GPs** and YM. Data are presented as mean ± SD (n = 3).

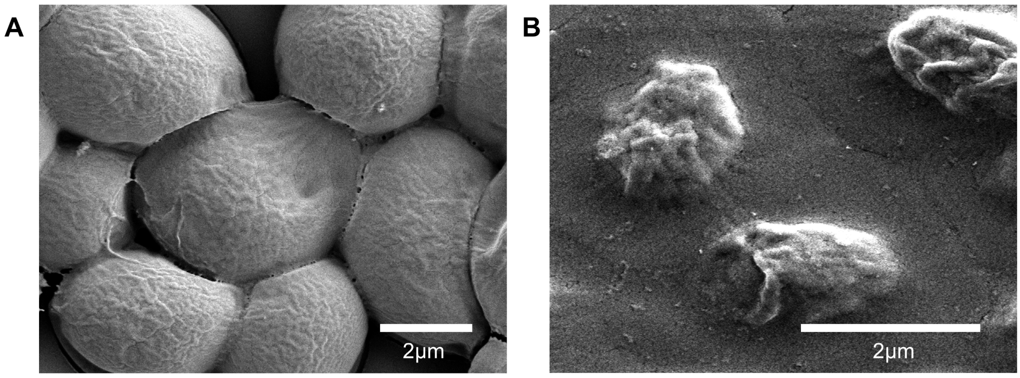

Figure S14. (A) SEM of yeast. (B) SEM of GPs.

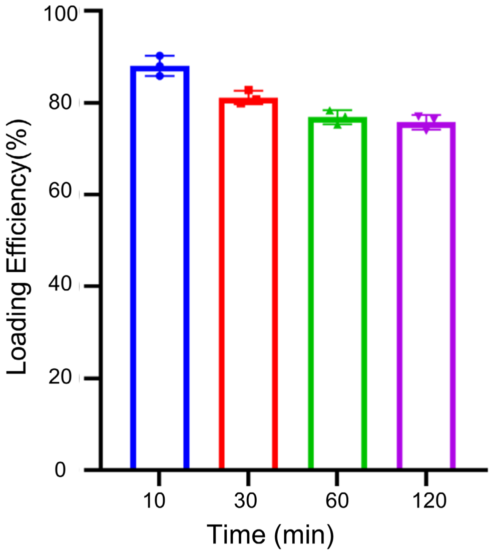

Figure S15. Acid stability of MTBPB/GPs evaluated by loading efficiency under simulated gastric conditions (pH 1.2) at different time points (10, 30, 60, and 120 min). Data are presented as mean ± SD (n = 3).

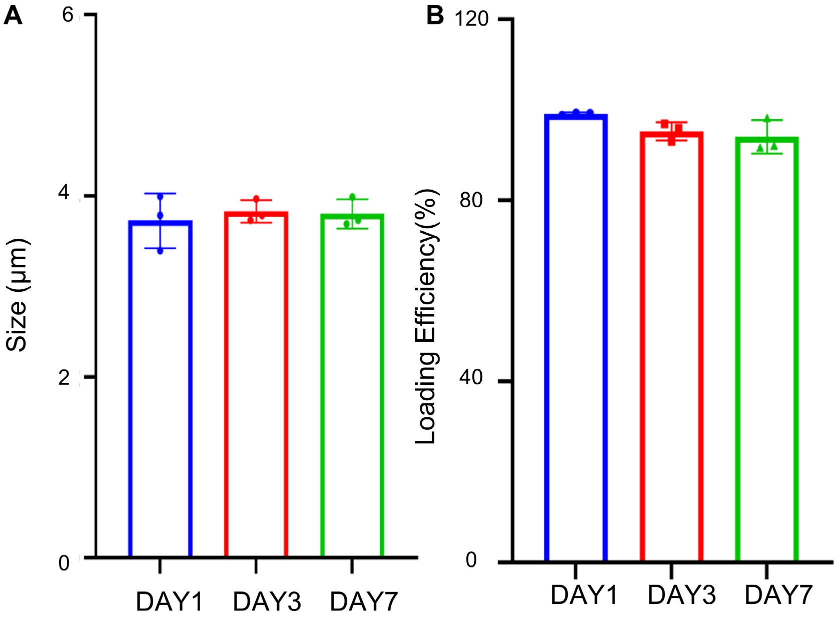

Figure S16. The (A) particle size and (B) loading efficiency of **MTBPB/GPs** after incubation for different periods of time. Data are presented as mean ± SD (n = 3).

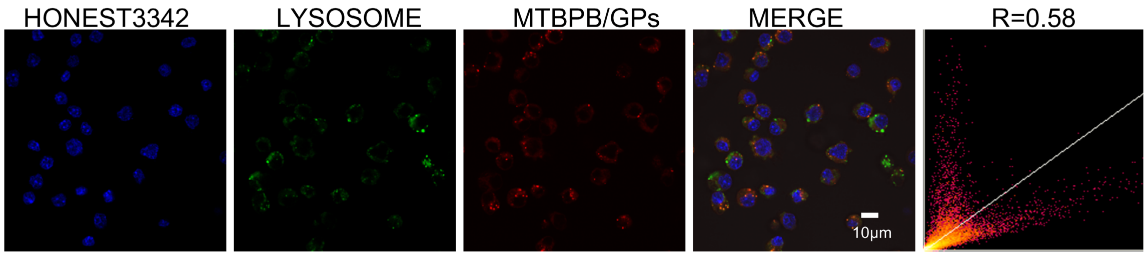

Figure S17. Confocal microscopy images showing the intracellular distribution of **MTBPB/GPs** (red) and lysosomes (green) in LPS-stimulated M1 macrophages. Nuclei were stained with Honest 3342 (blue). Quantitative colocalization analysis yielded a Manders' coefficient (R) of 0.58.

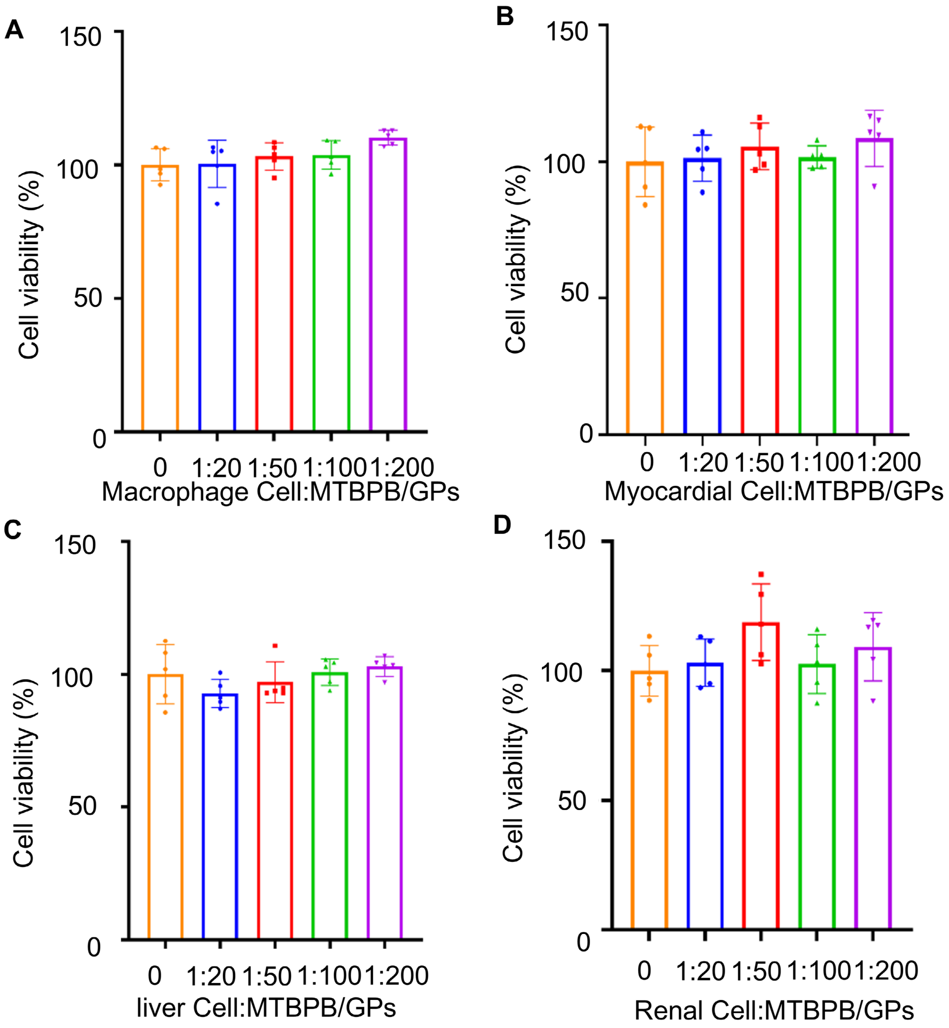

Figure S18. Effects of **MTBPB/GPs** on the proliferation activity of cells. (A) The effect of different concentrations of **MTBPB/GPs** on the proliferation activity of macrophages (RAW264.7), (B) myocardial cells (H9C2), (C) live cells (AML12) and (D) renal cells (293T). Data are presented as mean ± SD (n = 5).

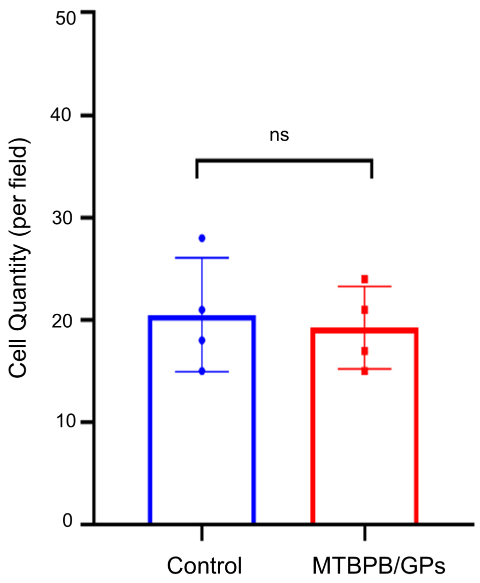

Figure S19. Quantitative analysis of the effect of **MTBPB/GPs** on macrophage migration after 24h of oral administration. Data are presented as mean ± SD (n = 4), ns= no significance.

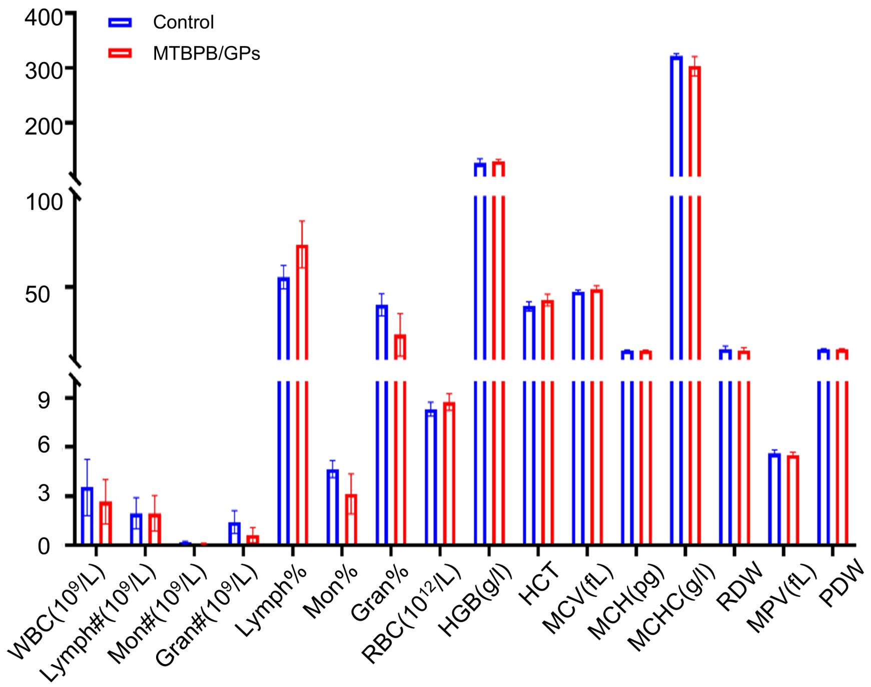

Figure S20. Hematological parameters of whole blood of healthy mice before and after 7 days of oral administration **MTBPB/GPs.** Data are presented as mean ± SD (n = 5).

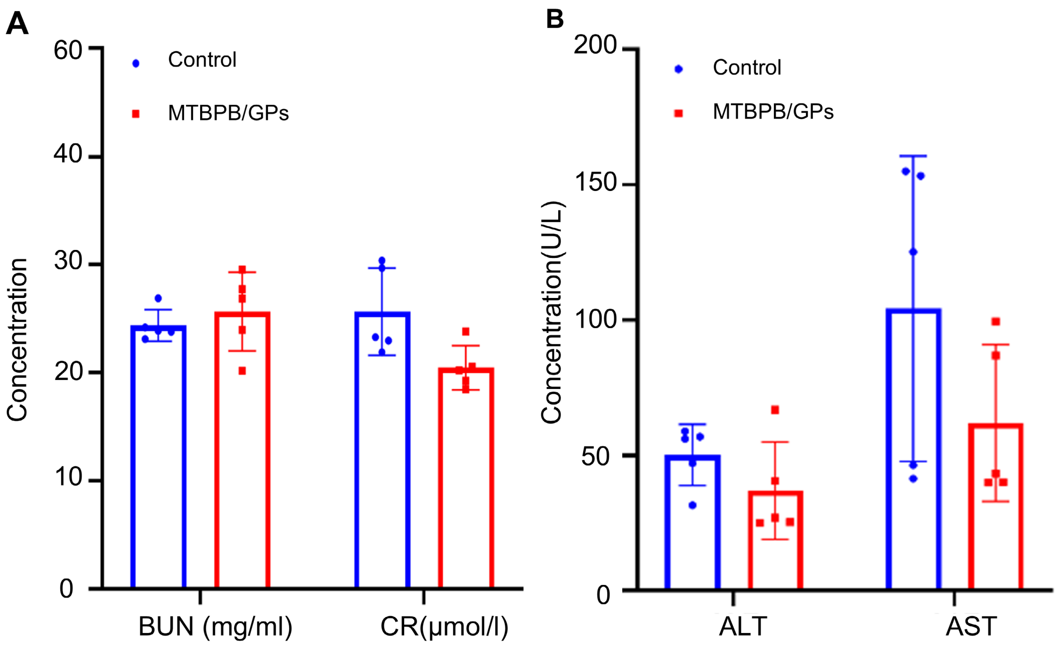

Figure S21. In vivo liver and kidney functional markers examined in the peripheral blood of healthy mice before and after 7 days of oral administration of **MTBPB/GPs**. (A) Serum levels of blood urea nitrogen (BUN) and creatinine (CR) and (B) aspartate transaminase (AST) and alanine aminotransferase (ALT). Data are presented as mean ± SD (n = 5).

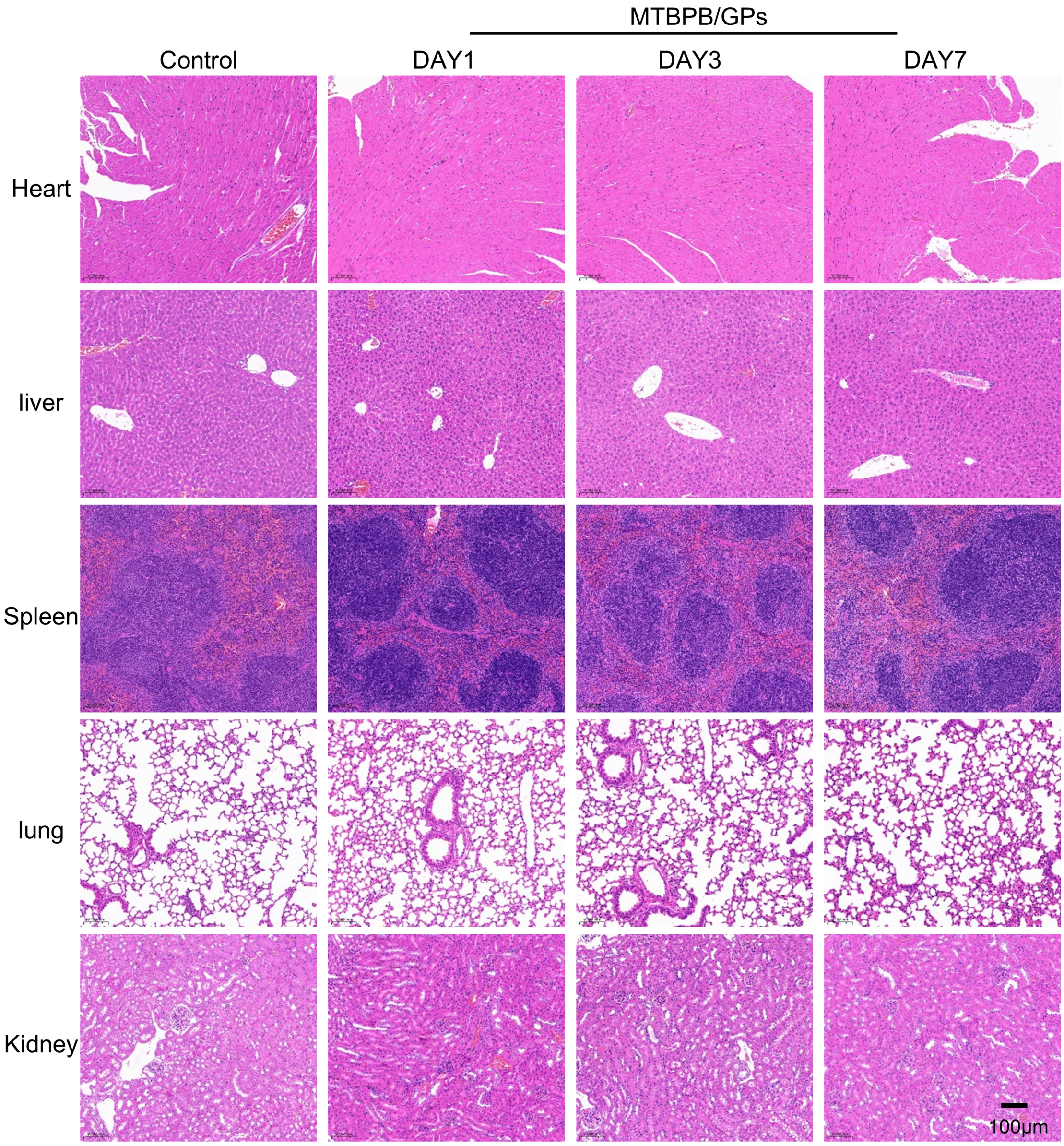

Figure S22. H&E staining of major organs including heart, lungs, liver, spleen and kidneys in healthy mice at different time points (day1, 3, 7) of oral administration of **MTBPB/GPs.** Data are presented as mean ± SD (n = 5).

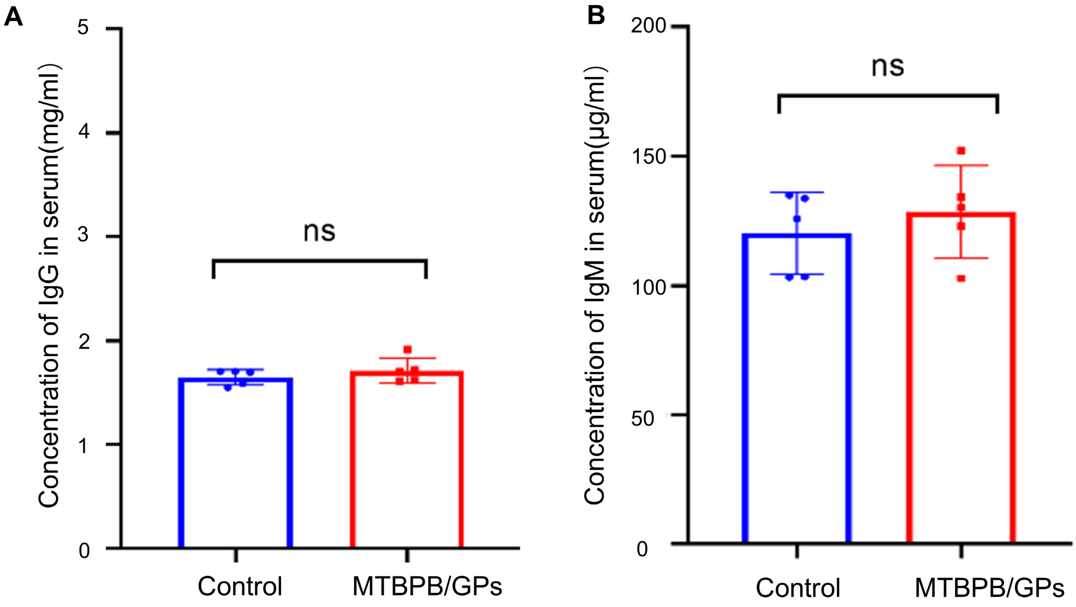

Figure S23. Immunogenicity of **MTBPB/GPs**. Healthy mice were orally administrated **MTBPB/GPs** before and after 7 days, the level of (A) IgG and (B) IgM in serum. Data are presented as mean ± SD (n = 5), ns= no significance.

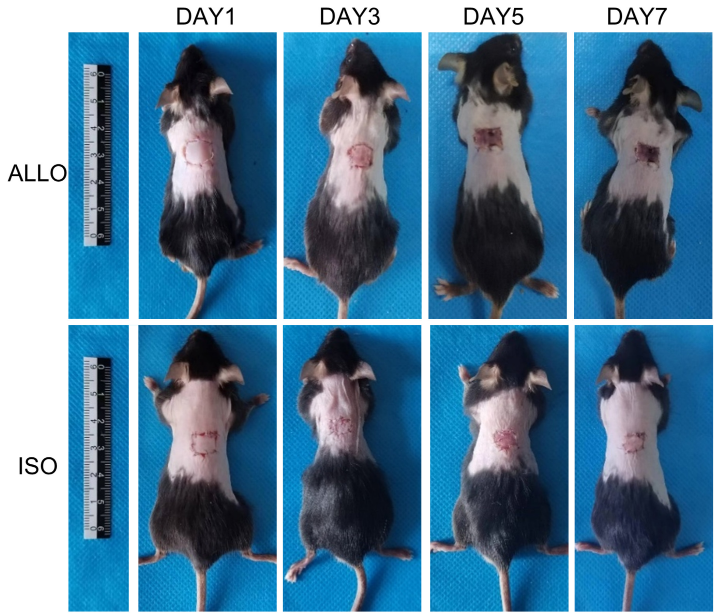

Figure S24. Representative pictures of 1, 3, 5, and 7 days of allograft and isograft mice.

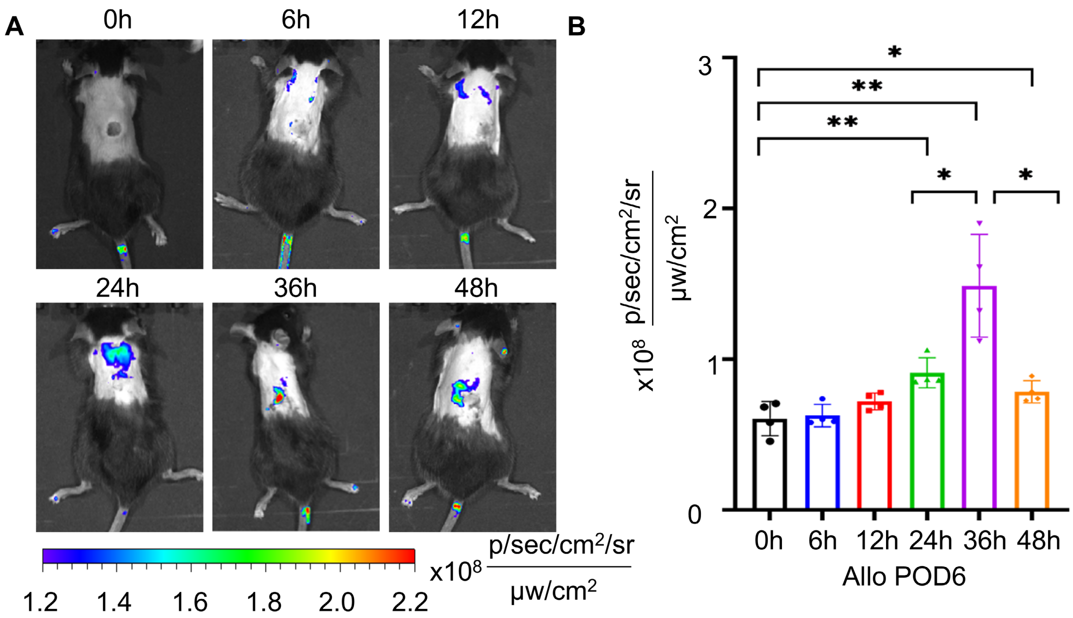

Figure S25. In vivo imaging of allograft mice on POD6 (A) Fluorescence imaging after 0, 6, 12, 24, 36 and 48 h after oral administration of **MTBPB/GPs**. (B) Quantified fluorescence intensity (FI) of skin grafts. Data is presented as mean ± SD (n = 4), $^{*}p < 0.05$, $^{**}p < 0.01$.

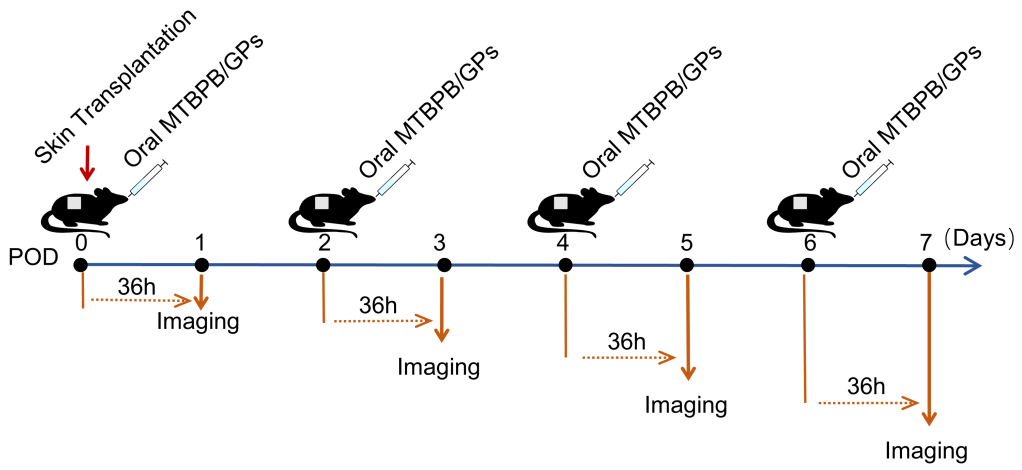

Figure S26. Different timelines for oral **MTBPB/GPs** and in vivo imaging.

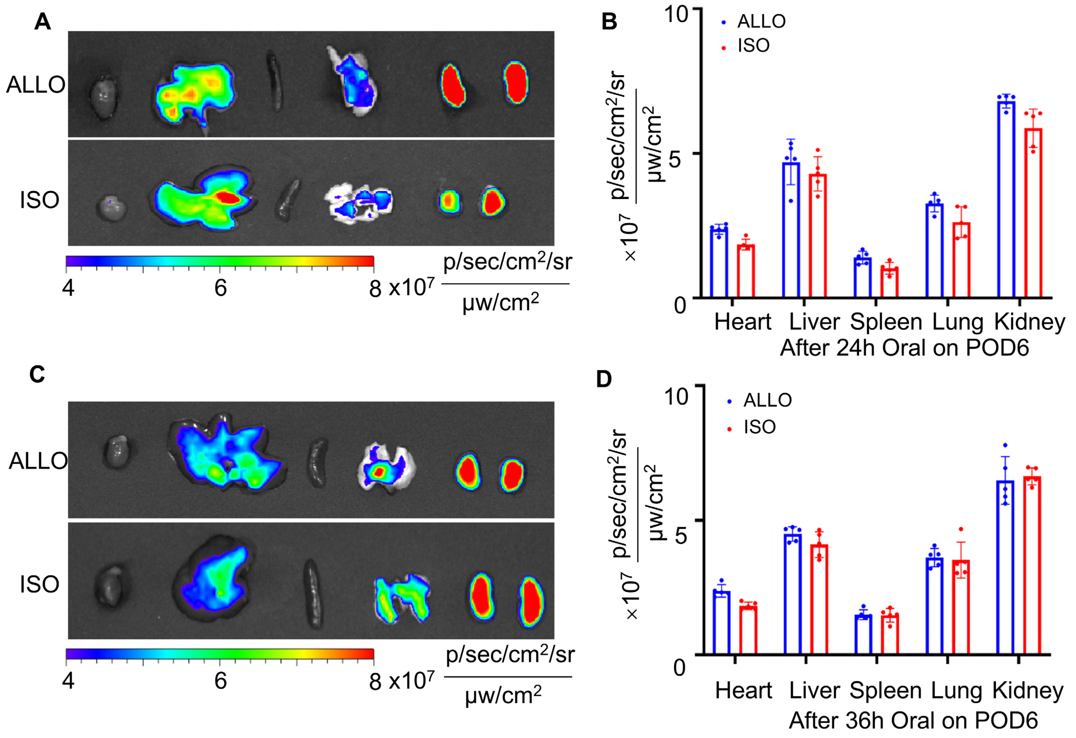

Figure S27. Tissue distribution of allograft and isograft mice after oral **MTBPB/GPs**. (A) Representative ex vivo fluorescence image of major organs after oral **MTBPB/GPs** for 24 h on POD6 and (B) quantitative fluorescence intensity plot (n = 5). (C) Representative ex vivo fluorescence image of major organs after oral **MTBPB/GPs** for 36 h on POD6. (D) Biodistribution of **MTBPB/GPs** in different organs. Data are presented as mean ± SD (n = 5). The organs were imaged with Perkin Elmer *IVIS Lumina* imaging system (Ex/Em = 480/620 nm).

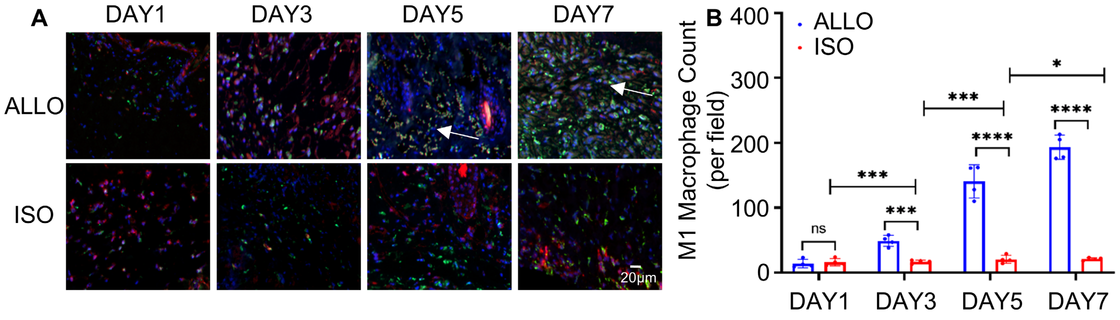

Figure S28. (A) Immunofluorescence staining of CD68^{+} iNOS^{+} M1 macrophages in allografted skin at POD 1, 3, 5, and 7; FITC-labeled for staining CD68, Cy3-labeled for staining iNOS, nuclei were stained with DAPI. (B) Quantification of M1 macrophage infiltrates in allografted skin. Data are presented as mean ± SD (n = 5).

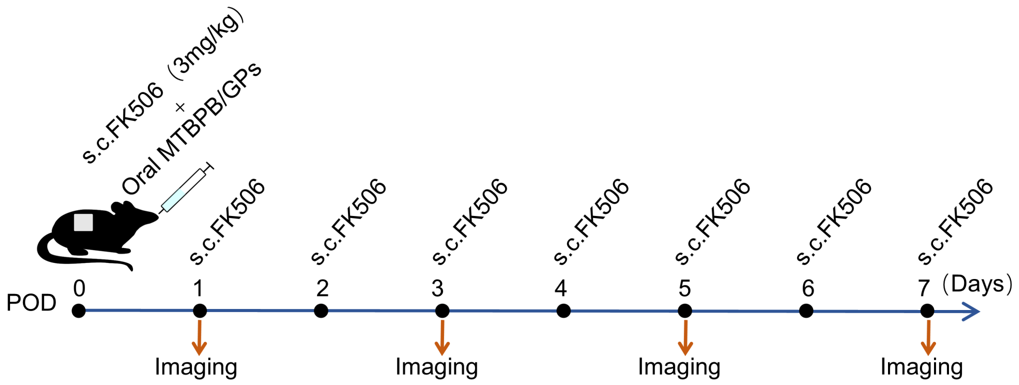

Figure S29. Different timelines of FK506 treatment followed by oral **MTBPB/GPs** in vivo imaging.

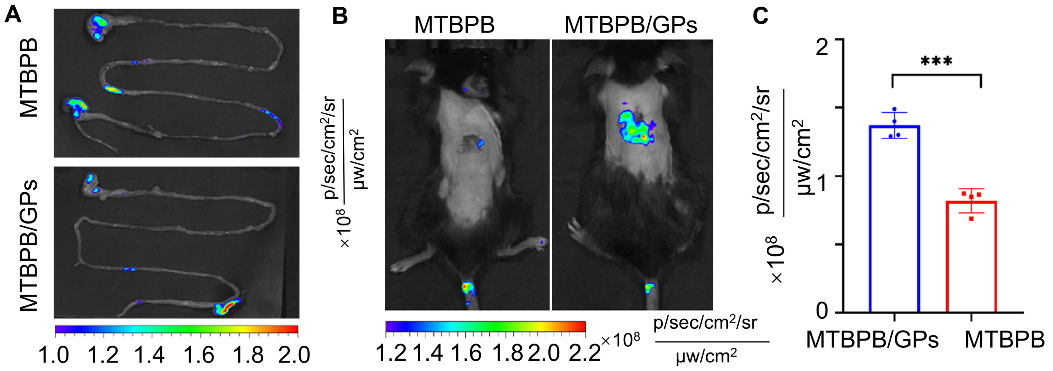

Figure S30. In vivo and ex vivo fluorescence imaging. (A) Ex vivo fluorescence signal of GI tract excised from the allograft mice orally administrated with **MTBPB/GPs**, **MTBPB** for 36h. (B) Fluorescent imaging of allograft mice (POD 6) after oral administration of **MTBPB/GPs**, **MTBPB** for 36 h. (C) Evolution of the signal intensity in the grafted skin (n = 4). The organs were imaged with Perkin Elmer *IVIS Lumina* imaging system (E_x/E_m = 480/620 nm). $^{***}P < 0.001$

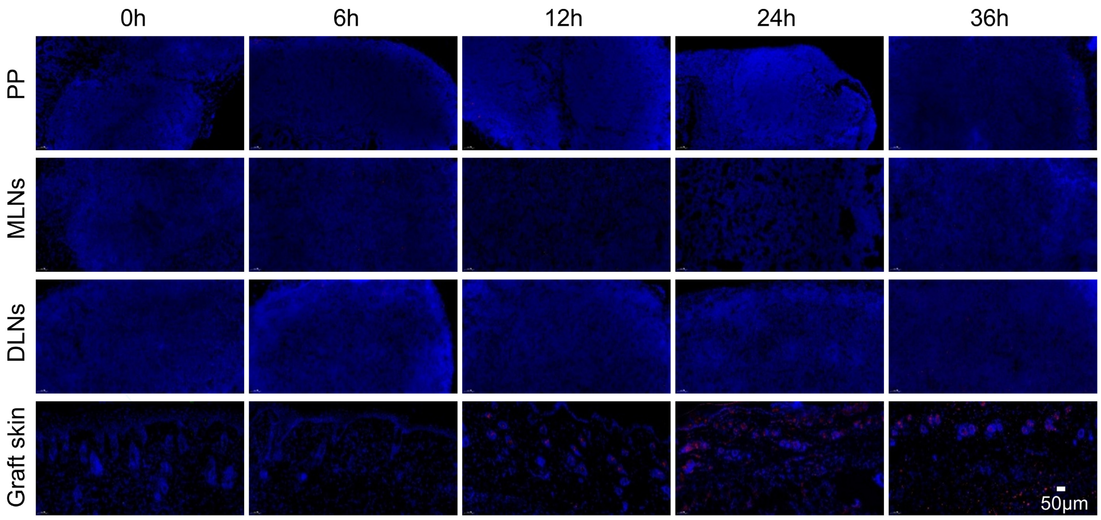

Figure S31. Fluorescence images of sections of lymphoid tissues (PP, MLNs, DLNs) and the section of grafted skin at indicated time points after oral **MTBPB/GPs** in allograft mice.
